# Telehealth Interventions for Family Caregivers of Persons with Chronic Health Conditions: A Systematic Review of Randomized Controlled Trials

**DOI:** 10.1155/2021/3518050

**Published:** 2021-05-21

**Authors:** Lucinda J. Graven, Robert L. Glueckauf, Rachel A. Regal, Nancy K. Merbitz, Mia L. A. Lustria, Brittny A. James

**Affiliations:** ^1^Florida State University College of Nursing, Tallahassee, FL, USA; ^2^Florida State University College of Medicine, Tallahassee, FL, USA; ^3^Virginia Commonwealth University, Richmond, VA, USA; ^4^Louis Stokes Cleveland Veterans Administration Medical Center, Cleveland, OH, USA; ^5^Florida State University College of Communication and Information, Tallahassee, FL, USA; ^6^Georgia Gwinnett College, Lawrenceville, GA, USA

## Abstract

**Objective:**

The purpose of this study was to provide an in-depth analysis of the components and outcomes of telehealth interventions for family caregivers of individuals with chronic health conditions.

**Methods:**

A systematic review of 17 databases was conducted for randomized controlled trials published between January 2002 and January 2017. Interventions were analyzed based on type of telecommunication modality, caregiver and care recipient characteristics, intervention components, and caregiver outcomes.

**Results:**

A total of 57 articles met criteria for inclusion. Telephone was the most frequently used mode of telehealth delivery and focused primarily on caregivers of older adults with dementia and stroke. Skills training was the most prevalent treatment strategy across telephone, web, and combined telephone and web modalities. Improved psychological functioning was reported most frequently across telehealth modalities.

**Conclusion:**

Telehealth is an effective tool in delivering caregiver interventions and leads to significant improvement in caregiver outcomes. Telephone was used most often to deliver cognitive-behavioral and psychoeducational strategies as compared to web and combined telephone and web modalities. Further research is needed to examine the effects of telehealth interventions on caregiving skills and self-efficacy, as well as health outcomes.

## 1. Introduction

Approximately 44 million family members and significant others residing in the United States provide unpaid assistive care to children and adults with chronic health conditions [1]. The responsibilities of family caregivers vary considerably depending on the age of the care recipient, severity of the disability, and availability of resources [2]. As a consequence of caregiving activities (e.g., providing assistance with bathing and managing medications) and other responsibilities (e.g., work commitments), caregivers must perform a difficult balancing act. It is commonplace for family caregivers to prioritize the needs of their loved ones with chronic illnesses above their own personal concerns and lifestyle preferences. Although they often verbalize the importance of health-promoting activities, many caregivers find it challenging to create time for self-care opportunities, to attend doctors' appointments, and to ask for caregiving assistance from other family members [3, 4]. Caregivers often report substantial declines in social activities, disrupted familial relationships, financial problems, and deterioration in physical and mental health [5].

In an effort to mitigate these difficulties, governmental and healthcare organizations have called for research on the effects of telehealth interventions in meeting the informational, psychosocial, and healthcare needs of family caregivers. Several of these entities have emphasized the importance of ensuring ease of access, uptake of services, and cost-effectiveness [6–8]. Telehealth services for family caregivers facilitate access to interventions in convenient locations and in turn reduce costs of transportation and respite care. Caregivers with part- or full-time employment also are able to receive needed services during off-hours, thus reducing work disruptions and potential loss of wages. Furthermore, caregivers residing in rural areas or crowded urban communities may find telehealth modalities more convenient, reducing travel costs, parking restrictions, and/or risks to self and property [9, 10].

Although research on the outcomes of telehealth interventions with family caregivers has been slower in evolving compared to research focusing on individuals with chronic illnesses, this trend has changed markedly over the past decade. Prior systematic reviews [2, 11] on telehealth intervention provided useful background information about caregiver and care recipient characteristics, types of telecommunication modalities and intervention approaches, and their overall pattern of findings. These reviews supported the effectiveness of telehealth interventions in improving the emotional and psychosocial functioning of family caregivers of persons with chronic health conditions. Although these reviews [2, 11] represent important initial efforts in evaluating the outcomes of caregiver telehealth interventions, a notable shortcoming limited the validity of their findings. Both reviews [2, 11] incorporated studies with nonexperimental research designs, ranging from case studies to quasi-experiments, thus weakening the causal conclusions that could be drawn about the efficacy of telehealth caregiver interventions.

To address this limitation, this review examined the results of randomized clinical trials assessing the effects of telehealth interventions on the psychosocial functioning, health status, and caregiving abilities of family caregivers. The decision to focus on the results of randomized controlled trials was based on prevailing standards for judging treatment efficacy [12]. Special attention also was given to the types of telecommunication modalities deployed in delivering caregiver telehealth interventions, user applications and options incorporated during the course of treatment, and their relationship to caregiver outcomes.

## 2. Methods

### 2.1. Identification of Studies

A literature search was performed to identify studies examining the efficacy of randomized controlled trials of telehealth interventions for family caregivers of individuals with chronic conditions published in English-language peer-reviewed journals from January 2002 to January 2017. Seventeen interdisciplinary scholarly databases were searched (Table 1) using the following terms in various combinations: “caregiver,” “telemedicine,” “telehealth,” “video,” “computer,” “Internet,” “conference,” “remote consultation,” “train,” “intervention,” “randomized,” “controlled,” and “clinical trial.” These keywords were selected based on their capacity to retrieve a wide range of pertinent articles and to ensure identification of new articles, as well as those not fully indexed. Strict inclusion/exclusion criteria also were adopted to enhance the relevance of articles for study inclusion (Table 2).

**Figure 1:**
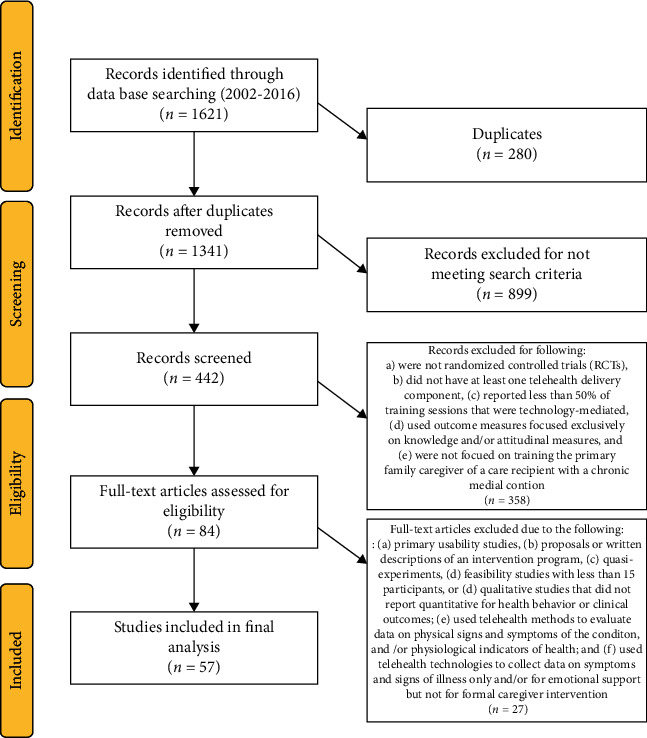
Prisma diagram.

The initial search yielded 1621 potentially relevant studies archived for further review. After the removal of duplicate articles (*n* = 280), the remaining articles (*n* = 1341) were screened for eligibility. Ineligible studies were excluded (*n* = 899) (e.g., not written in English, feasibility studies, quasi-experimental design, and qualitative studies). Abstracts of the remaining studies (*n* = 442) underwent further evaluation, with additional studies excluded (*n* = 358) due to failure in meeting the inclusion criteria. Next, the full texts of the remaining articles (*n* = 84) were reviewed by two independent coders to confirm eligibility, resulting in the exclusion of 27 additional articles. A total of 57 articles met all inclusion criteria and were included in this systematic review (Figure 1). Due to the heterogeneity of study populations, interventions, and outcomes examined, we opted not to pursue a formal meta-analysis of the included studies.

### 2.2. Identification and Operationalization of Intervention Characteristics

Each study was examined to identify telecommunication modalities used during treatment, caregiver and care recipient sample characteristics, outcome measures, intervention characteristics, and primary findings. Description of these key characteristics, organized by delivery modality, can be found in Tables 3–5 (telephone, web, and combination web and telephone interventions). The interventions were further inspected to classify intervention components, caregiver applications, and user control (Table 6). Caregiver intervention components consisted of group or individual skills training, psychoeducational and resource materials, self-monitoring/tracking, reminders, and group or individual counseling. Caregiver applications included the use of email, discussion forum/online chat, online journaling, text messaging, telephone calls (independent of intervention), and provision of useful links and resources. Regarding user control, interventions were categorized as either facilitator-guided or self-guided. Facilitator-guided interventions used healthcare professionals to lead sessions or maintain the flow of participation during the intervention. Self-guided interventions relied exclusively on participants to complete all phases of the program.

### 2.3. Coding of Intervention Characteristics

Two researchers coded eligible studies independently on caregiver and care recipient demographics, intervention design, telehealth modalities utilized, methodological characteristics, and study outcomes. Operational definitions were summarized in a codebook to ensure coding procedures were performed consistently and accurately. Coder agreement was assessed on quantifiable factors across a subset of categories. Findings from both reviewers were compared, and areas of disagreement were discussed until consensus was achieved.

### 2.4. Data Analytic Strategy

Analysis of systematic review findings was organized based on the primary telecommunication modalities used in delivering caregiver interventions. Studies were placed into three different categories: (a) telephone interventions, (b) web interventions, and (c) combination of telephone and web interventions. This data analytic strategy was implemented due to limited variation in caregiver populations. Over half of included studies targeted family caregivers of older adults with dementia or stroke. Two advantages emerged from this approach: (a) optimal differentiation across caregiver telehealth outcome studies and (b) substantive methodological and conceptual comparisons. Only a small percentage of included studies reported caregiver-care recipient dyadic results. Thus, this review addressed caregiver outcomes only.

Data analysis focused primarily on counts and percentages across the major elements of the review (i.e., characteristics of caregiver samples, intervention strategies, outcomes, and caregiver applications and user controls). First, descriptive statistics on caregiver sample characteristics were calculated for both the overall sample of caregivers and separately for each telecommunication delivery mode. Similarly, the number and percentage of specific types of intervention strategies were assessed for the overall sample of studies and separately for each of the three categories of delivery modalities.

Second, vote counting (i.e., number and percentage) of statistically significant caregiver target outcomes was conducted for both the overall sample and separately for each of the three telecommunication modalities. The term, improved outcome, was used only if the target outcome of the intervention group was statistically significant compared to the comparison/control groups (Tables 3–5). Target outcomes included psychological well-being, physiological functioning, caregiving skills, social functioning, problem-solving/goal setting, and quality of life. Studies were included in this count once. The rationale for this procedure was to facilitate accurate and consistent percentage calculations of the total number of significant studies versus the total number of studies and for each of the three modality categories.

Last, the number and percentage of caregiver applications and user control features were assessed for both the overall sample and separately for each of the three telecommunication modalities (Table 6).

## 3. Results

### 3.1. Characteristics of Telehealth Randomized Controlled Trials across Modalities

#### 3.1.1. Samples, Journal Outlets, and Study Locations

A total of 57 randomized controlled trials evaluating the efficacy of telehealth interventions for caregivers of persons with chronic health conditions were included in this systematic review (Figure 1). Tables 3–5 summarize the key characteristics and outcomes of these studies, including telehealth delivery modalities, type of caregiver sample, primary outcome measures, intervention description, and key findings. Of the 57 included articles, only 16 (28%) were caregiver-care recipient dyad studies. The majority of intervention studies (*n* = 32, 56%) centered on family caregivers of older adults with neurological conditions, such as dementia and stroke. A substantially smaller percentage of studies focused on interventions for caregivers of persons with cancer (*n* = 6, 11%) and caregivers of children, adolescents, and young adults with chronic health conditions (*n* = 6, 11%). Only a few intervention studies targeted caregivers of persons with heart failure (*n* = 2, 4%) and persons receiving hospice or palliative care (*n* = 2, 4%).

Of the 57 included studies, 58% (*n* = 33) were telephone-mediated, 25% (*n* = 14) used web technologies, and the remainder (*n* = 10, 17%) used a combination of telephone and web modalities (Tables 3–5). Last, the 57 included studies were published in 42 different journals, primarily psychological and medical in nature. The randomized controlled trials were conducted in nine different countries, with the United States accounting for 80% of publications.

#### 3.1.2. Intervention Components

Of the 57 studies, the majority of interventions (*n* = 37, 65%) were ≤ six months long, included participant follow-up for ≤ six months (*n* = 17, 30%), and did not incorporate booster sessions (*n* = 53, 93%). Furthermore, most studies (*n* = 47, 82%) did not collect postintervention data. Across the 57 studies, interventions included at least one of five different components, including skills training (group, individual, or both), psychoeducational and resource materials, self-monitoring/tracking, reminders, and counseling (group or individual). Skills training was conducted most often (*n* = 43, 75%) either individually (*n* = 32, 56%), in a group (*n* = 9, 16%), or using a combination of individual and group training (*n* = 2, 4%). Topics covered in the skills-building sessions varied across telehealth modalities and included training on effective communication, disease-specific caregiving skills, care recipient safety, social skills, and problem-solving. Psychoeducational and resources materials also were commonly used (*n* = 40, 70%) to supplement intervention components and covered similar topics across modalities (e.g., problem-solving, stress management, and coping skills). Other less frequently used strategies consisted of individual (*n* = 8, 14%) or group (*n* = 7, 12%) counseling and the use of self-monitoring/tracking (*n* = 14, 25%) and reminders (*n* = 4, 7%) (Table 6).

#### 3.1.3. Outcomes

Forty-two of 57 studies (74%) reported significantly greater improvement on at least one target outcome for caregivers participating in the intervention versus those in the other groups. Fifteen of the 57 studies (26%) reported nonsignificant findings on target outcomes (Tables 3–5). Significant differential improvement in psychological well-being was noted in the intervention conditions for the majority of studies (*n* = 36, 63%), followed by enhanced health and physiological functioning (*n* = 8, 14%), as well as improved caregiving skills (*n* = 8, 14%). Less common findings included significant improvements in social functioning (*n* = 6, 11%), quality of life (*n* = 5, 9%), and problem-solving/goal setting (*n* = 3, 5%) for the intervention groups as compared to comparison/control group counterparts. Fifteen of the 57 (26%) included studies did not show significant improvement in caregiver outcomes following intervention compared to the comparison/control groups (Tables 3–5).

#### 3.1.4. Applications and User Control

Across the 57 studies, 12 (21%) included useful links and resources for caregivers to supplement the intervention, such as psychological and physical symptom inventories [48], caregiving tip sheets [54], online educational videos/modules [54, 56], and links to disease-specific websites [58]. Discussion forums/online chats were used in nine studies (16%) to foster communication and peer support [11, 49, 51, 52, 56, 60, 61, 63, 67]. Telephone calls independent of the intervention also were used in nine studies (16%) to provide support [21, 39] and to facilitate consultation and follow-up [30, 35, 40, 54, 62, 65, 67]. Alternately, email was used in seven studies (12%) to maintain communication with participants [48, 52, 54, 56, 61, 65] and to distribute materials to caregivers [47]. Only one study (2%) included online journaling to facilitate caregivers' documentation of caregiving issues and care recipients' health status [67] (Table 6).

### 3.2. Telephone Interventions

#### 3.2.1. Samples

Of the 33 telephone intervention studies, 23 (70%) included caregivers only, whereas 10 (30%) engaged both caregivers and care recipients in dyadic intervention. Sixteen studies (48%) included caregivers of older adults with progressive dementia, followed by caregivers of individuals with stroke (*n* = 4, 12%) and cancer (*n* = 4, 12%). Caregivers of individuals with unspecified chronic conditions (*n* = 3, 9%), neurological injuries (i.e., spinal cord and traumatic brain injury; *n* = 2, 6%), cardiovascular conditions (*n* = 2, 6%), and depression (*n* = 1, 3%), as well as adults in hospice care (*n* = 1, 3%), were less represented. Fifteen studies (45%) included more than 100 participants, 13 studies (39%) included 50-100 participants, and five studies (15%) included 10–50 participants (Table 3).

#### 3.2.2. Intervention Components

Among the five types of components implemented in the 33 caregiver telephone interventions, skills training procedures (*n* = 27, 82%) were deployed most often, the bulk of which were delivered to individual caregivers (*n* = 22, 67%), followed by the use of group format (*n* = 3, 9%) or a combination of group and individual training (*n* = 2, 6%). The foci of skills training included communication enhancement techniques, disease-specific caregiving methods, monitoring and management of care recipient's physical and psychological well-being, care recipient safety, and fostering caregiving self-care activities. A variety of materials were used to bolster skills training, including print materials (e.g., pamphlets, tip sheets [14, 15], and caregiver workbooks [27, 38]) and video modules/DVDs [27, 33]. Psychoeducational and resource information also were commonly used (*n* = 20, 61%), primarily in conjunction with problem-solving strategies, stress management, relaxation, and coping skills techniques. Group or individual supportive counseling (*n* = 10, 30%), caregiver self-monitoring/tracking (*n* = 7, 21%), and reminders (*n* = 1, 3%) were incorporated less frequently in the telephone interventions (Table 6).

#### 3.2.3. Outcomes

Twenty-one of 33 telephone studies (64%) reported statistically significant caregiver improvements on target outcomes in the intervention conditions compared to little or no change in comparison/control conditions. Of the 33 telephone studies, 12 (36%) reported nonsignificant intervention effects on target outcomes (Table 3). Differential posttreatment gains were found across the following sets of variables in descending order of frequency: (a) psychological well-being (e.g., decreased depression and anxiety; improved coping) (*n* = 16, 48%), (b) enhanced health and physiological functioning (increased physical activity, decreased blood pressure) (*n* = 6, 18%), (c) improved caregiving skills (*n* = 4, 12%), (d) increased social functioning (*n* = 4, 12%), (e) enhanced problem-solving/goal setting (*n* = 3, 9%), and (f) improved quality of life (*n* = 3, 9%). In contrast, 12 studies (36%) did not obtain significantly greater gains in caregiver outcomes for the intervention groups versus the comparison/control groups (Table 3).

#### 3.2.4. Applications and User Control

Five studies (15%) included telephone calls independent of the intervention to provide real-time support [21, 39], consultation, and follow-up [30, 35, 40]. Ninety-four percent of telephone-mediated studies (*n* = 31) were facilitator-guided, whereas only 6% (*n* = 2) used a self-guided approach (Table 6).

### 3.3. Web Interventions

#### 3.3.1. Samples

Of the 14 web studies, nine (64%) included family caregivers only, focusing on caregivers of adults with dementia (*n* = 4, 44%), children/adolescents with traumatic brain injury (*n* = 2, 22%), adults with neurodegenerative disorders (*n* = 1, 11%), stroke (*n* = 1, 11%), and unspecified chronic illness (*n* = 1, 11%). Five studies (36%) engaged both caregiver and care recipient dyads and involved caregivers of children/adolescents with traumatic brain injury (*n* = 2, 40%) and attention deficit-hyperactivity disorder (*n* = 1, 20%), as well as caregivers of adults with advanced stage cancer (*n* = 1, 20%) and stroke (*n* = 1, 20%). Seven studies (50%) recruited over 100 participants, three studies (21%) had 50-100 participants, and four studies (29%) included 10-50 participants (Table 4).

#### 3.3.2. Intervention Components

Skills training (group or individual), psychoeducational and resource materials, group counseling, self-monitoring/tracking, and reminders were the primary intervention components used in the web interventions. Skills training (*n* = 11, 79%) consisted of both group (*n* = 6, 43%) and individual formats (*n* = 5, 38%) and covered topics such as social skills enhancement (e.g., arranging care assistance [47]), assertiveness, disease-specific caregiving skills [48, 56], and effective communication with care recipients and healthcare professionals [53]. Psychoeducational and resource materials (*n* = 11, 79%) included information about coping skills, problem-solving, relaxation techniques, stress management, and/or cognitive behavioral training/restructuring. Self-monitoring/tracking (*n* = 2, 14%), reminders (*n* = 2, 14%), and group counseling (*n* = 2, 14%) were used infrequently in the web studies (Table 6).

#### 3.3.3. Outcomes

Eleven of 14 (79%) web studies reported significant improvements on target outcomes in the intervention groups as compared to little or no change in the comparison/control groups. Three (21%) of 14 web studies reported nonsignificant intervention effects on target outcomes (Table 4). Differential increases in psychological well-being (e.g., decreases in depression, anxiety, and stress) were the predominant outcomes in the intervention arms across the 11 studies (79%). Other significant outcomes were obtained for caregiving skills (i.e., improved caregiving self-efficacy; *n* = 1, 7%) and quality of life (i.e., increased positive appraisals of caregiving; *n* = 1, 7%; improved quality of life; *n* = 1, 7%) (Table 4).

#### 3.3.4. Applications and Use Control

Eight studies (57%) provided useful web links and resources (e.g., online educational materials, tip sheets [54] and asynchronous modules [55]) to supplement the intervention. Email was incorporated in five studies (36%) to enhance communication with the research team [48, 52, 54, 56] and to provide materials to participants [47]. Five studies (36%) used discussion forums/online chat to facilitate communication and to encourage peer support [48, 49, 51, 52, 56]. Telephone calls independent of the intervention were used to follow up with participants in one study (7% [54]). Eight of the web interventions were self-guided (57%) and six (43%) were facilitator-guided (Table 6).

### 3.4. Combined Telephone and Web Interventions

#### 3.4.1. Samples

Of the 10 combined telephone and web studies, 9 (90%) recruited family caregivers only and 1 (10%) involved both caregivers and care recipients in dyadic intervention. These studies included caregivers of adults with a variety of chronic medical conditions, including progressive dementia (*n* = 5, 50%), heart failure (*n* = 1, 10%), traumatic brain injury (*n* = 1, 10%), cancer (*n* = 1, 10%), anorexia nervosa (*n* = 1, 10%), and unspecified older individuals receiving hospice care (*n* = 1, 10%). Seven studies (70%) recruited over 100 participants and three studies (30%) included between 50 and 100 participants (Table 5).

#### 3.4.2. Intervention Components

The primary intervention components included psychoeducational and resource materials, individual skills training, self-monitoring/tracking, counseling (group or individual), and reminders. Psychoeducational and resource materials, providing information about problem-solving, modification of cognitions and behavior (e.g., goal setting and attainment [61]), stress management [68], and coping skills [69] were the predominant intervention components across the combined telephone and web studies (*n* = 9, 90%). Five studies (50%) incorporated individual skills training focusing on disease-specific caregiving strategies (e.g., video vignettes on dementia progression [62]), problem-specific response skills [66], and event scheduling/time management [67, 68]. Self-monitoring/tracking (*n* = 5, 50%) was used to follow caregivers' progress and completion of intervention-related components. Strategies, such as individual (*n* = 2, 20%) (e.g., weekly therapist guidance by phone/email [61]) and group (*n* = 1, 10%) counseling (e.g., discussion of caregiver problems and identification of helpful resources [60]), as well as reminders (*n* = 1, 10%), were used infrequently in the combined interventions (Table 4).

#### 3.4.3. Outcomes

All ten combined telephone and web studies (100%) reported significant caregiver improvements on target outcomes in the intervention conditions as compared to little or no change in the comparison/control conditions (Table 5). Improvements in psychological well-being (e.g., decreased depression and stress; increased coping) in the intervention conditions were reported across 9 of 10 studies (90%). Enhanced social/family functioning (e.g., increased perceived social support) also was found among intervention participants in two studies (20%). Significant gains in caregiving skills (e.g., increased caregiver self-efficacy) were reported by intervention caregivers in two studies (20%). Similarly, greater improvement in well-being (e.g., increased participation in self-care activities) also was obtained in the intervention conditions as compared to comparison/controls counterparts in one study (10%). Finally, improvement in quality of life was found among intervention participants for 1 of 10 studies (10%) (Table 5).

#### 3.4.4. Applications and Use Control

Discussion forums/online chat were used in four studies (40%) to foster communication and provide support [60, 61, 63, 67]. Four studies (40%) provided useful web links and resources to supplement the intervention [60, 62, 65, 67]. Three studies (30%) included telephone calls with caregivers that were independent of the intervention [62, 65, 67], whereas two studies (20%) used email to communicate with caregivers [61, 65]. Online journaling was used in one study (10%) to facilitate documentation of psychological and physical challenges related to caregiving and care recipients' health [67]. Six studies (60%) were facilitator-guided and four (40%) were self-guided (Table 6).

## 4. Discussion

In examining the descriptive findings across modalities, telephone delivery was the most used approach. One possible explanation for the disproportionate use of telephone across studies lies in the demographic characteristics of caregivers of persons with dementia and stroke, who were more likely to be older adults and in turn, slower adopters of web technologies. In contrast, caregivers of children/adolescents were generally younger parents who incorporated web technologies into their daily lives and thus more likely to participate in web intervention. Of the six studies with caregivers of children, adolescents, and young adults, web modalities were used most often (*n* = 5, 83%), followed by combined telephone and web modalities (*n* = 1, 17%).

Next, over 70% of included studies focused exclusively on caregiver intervention rather than the caregiver-care recipient dyad. This decision may have been predicated by the diminished capacity of the care recipient to benefit from intervention, logistical issues, and limited exposure to dyadic treatment. First, over 50% of care recipients across the 57 studies were older adults with neurological disorders. Cognitive deficits, such as short-term memory loss and difficulties with verbal comprehension and expression, may pose obstacles in care recipient engagement in telehealth intervention [2, 11]. Second, logistic difficulties may have led to limited implementation of interventions involving both caregivers and care recipients. For example, organizing dyadic sessions may be problematic due to scheduling conflicts, competing job demands and collateral family obligations. Last, dyadic intervention requires specialized therapeutic skills many telehealth providers may not possess.

Regarding caregiver intervention strategies, skills training was used in 75% of studies across modalities. In contrast to previous research [11], both telephone (*n* = 27, 81%) and web approaches (*n* = 11, 78%) showed similar utilization rates in deploying skills training strategies. Prior research [11] reports disproportionately lower integration of skills training approaches in web interventions as compared to telephone modalities.

The format and methods of delivery of psychoeducational and resource materials varied considerably across modalities. In telephone interventions, caregivers received information booklets, manuals, or videos through the mail [14, 21, 27]. In web and combined telephone and web modalities, psychoeducation and resource materials were delivered online using modules, interactive platforms, and video vignettes [47, 48, 62]. Relatively high utilization rates of psychoeducation and resource information were noted across telephone, web, and combined telephone and web interventions (i.e., 61%, 79%, and 90%, respectively). This pattern was similar to that previously reported [11]. Overall, the technologies used in the interventions included in this review were basic, easy-to-use, and low-cost, such as telephone, videoconferencing [51, 58], DVD, web-based modules [33, 46, 50, 62], and interactive web-based platforms [47, 61]. However, one intervention incorporated a computer-mediated interactive voice response system [63] that required more sophisticated programming than other technologies highlighted in this review.

Self-monitoring/tracking was used infrequently across all modalities (*n* = 14, 25%). Limited incorporation of this component was difficult to interpret, particularly in light of its importance in guiding judgments about effectiveness of the treatment process. Furthermore, a more pronounced pattern of disuse was found for reminders. Only 7 of 57 studies included reminders to enhance attendance and adherence to intervention. A plausible explanation may be self-monitoring, and tracking systems were used in these studies, but not reported.

Next, caregiver applications were integrated into intervention for 21 of 57 studies (37%). Of the telephone interventions, only the independent phone call application was incorporated during intervention. However, the utilization rate was low (15%). Surprisingly, none of the telephone studies used text messaging. This finding may have its origin in lower adoption rates of digital technologies and/or use of text-based communication among caregivers of persons with dementia and stroke, a large proportion of whom were older adults.

In contrast, four applications were used in the web interventions and five with the combined telephone and web interventions (Table 6). Except for online journaling [67], both web and combined telephone and web interventions adopted similar caregiver applications. Useful links and resources were used in 57% of web applications and 40% of combined telephone and web interventions. Discussion forums were implemented in web and combined telephone and web interventions at similar rates (36% and 40%), respectively. In contrast, email was used more often in the web studies (36%) compared to combined telephone and web modalities (20%). This finding suggests telephone may have replaced email use in the latter telecommunication approach.

Last, a discrepant pattern for user control was noted across the three intervention modalities. Ninety-four percent of telephone and 60% of combined telephone and web interventions were facilitator-guided. In contrast, most web interventions (57%) were self-guided. Web technologies may be more appropriate for caregivers who are technologically savvy and require less guidance in assimilating information acquired through this modality.

Regarding intervention outcomes, significant improvements in at least one target caregiver outcome were found in 74% of the studies across all three modalities. Consistent with prior research [2], improvement in caregiver psychological well-being was the most frequently reported outcome (48%) in the telephone interventions. Significant improvements in psychological well-being also were obtained in the web (79%) and combined telephone and web interventions (90%), corroborating prior findings [11]. However, it is difficult to interpret the underlying reasons for increasing percentages of positive outcomes in psychological well-being across categories. This issue requires investigation in future studies.

Next, a discrepant pattern of results was found for physiological outcomes. Statistically significant improvements in health and physiological functioning were evidenced in both telephone (18%) and combined telephone and web methods (10%), but little or no change on these variables was found in the web interventions. This discrepancy may have been due to the self-guided nature of web modalities. In the absence of facilitator feedback, tailoring of intervention to bolster health and physiological functioning was precluded.

While this review provides an excellent summary of telehealth research through January 2017, this review does not capture more recent telehealth studies. However, since our review, there have been no published systemic reviews of telehealth interventions for caregivers of the chronically ill which encompasses a variety of study populations, interventions, and outcomes, as does this review. Further, although the current systematic review consisted of a variety of telehealth interventions, four major limitations were noted in the included studies. First, the overall pattern of intervention outcomes may have been skewed by the incorporation of telephone in two of three intervention categories. Although telecommunication technologies are increasing in popularity, a large population of caregivers continues to face considerable barriers in accessing interventions using these modalities, particularly older caregivers and those who reside rural geographical locations. Unmet technology training needs, high cost of equipment, and barriers in gaining access to Internet service, especially among older adult caregivers, may have accounted for the disproportionate use of telephone in caregiver telehealth studies [70]. Second, web and combined telephone and web interventions were conducted exclusively with caregivers of children, adolescents, and young adults. Telephone interventions were not performed with this caregiver population. Therefore, our knowledge of the efficacy of telephone interventions across the age continuum was restricted in this review. Third, although telehealth modalities hold considerable promise in increasing access to hard-to-reach populations, the majority of studies in this review did not specify caregivers' and care recipients' primary area of residence (e.g., rural or urban). This shortcoming limited evaluation of the impact of geographic location on treatment outcomes. Last, a large proportion of studies incorporated interventions to enhance caregiving and self-care skills. However, the impact of these activities on psychological functioning and health status as well as their level of implementation was not measured.

### 4.1. Research and Practice Implications

The overall findings of this review provided considerable support for the efficacy of telehealth interventions across a variety of psychosocial and emotional outcomes in diverse populations. A major strength of the current systematic review was the selection of studies using randomized controlled designs. This decision was predicated not only on the need for increased rigor in drawing causal conclusions but also on the growing use of telehealth interventions in caregiver research [2, 11].

Despite these encouraging developments, only a limited number of studies compared the effects of telehealth intervention versus an in-person comparator. For example, Davis et al. [18] compared the effects between telephone caregiver coping skills training and in-person intervention using the same protocol on changes in psychosocial functioning for dementia caregivers. The investigators found positive and equivalent gains in caregiver burden and distress at three months across both treatment conditions. In contrast, none of the web or combined telephone and web studies tested the differential impact of these modalities against similar in-person interventions. Thus, more research is needed to evaluate the efficacy of telehealth modalities versus in-person intervention delivery. Further, research is needed to test the equivalence or noninferiority of the effects of telecommunicated intervention versus traditional in-person interventions. The findings of such investigations will provide valuable information about the strengths and limitations of deploying specific telehealth delivery methods across different caregiver populations, caregiving challenges, and geographic and socioeconomic conditions.

This review found that telehealth modalities can be used successfully in delivering a variety of skills training and psychoeducational interventions for family caregivers. With recent advances in telehealth technologies, clinicians can conduct a wide variety of psychosocial and behavioral interventions that support caregivers' emotional functioning regardless of location and proximity to healthcare providers. Clinicians also have access to a wide array of online resources, videos, interactive modules, and other health-promoting technologies to enhance caregivers' skills in stress management, emotional well-being, and their health status. Although useful for all caregivers, telehealth modalities may be particularly beneficial in reaching rural caregivers, who are less likely to attend caregiving workshops due to financial or transportation issues.

Furthermore, telehealth intervention offers an effective method for promoting increased self-care in distressed caregivers. In a study by Piette et al. [65], caregivers received weekly, automated support calls with the option of obtaining guidance about care recipients' problems from an interdisciplinary team coupled with email suggestions for enhancing caregiver's self-care. Findings showed that caregivers who received the support calls reported significantly less caregiving strain compared to caregivers in the control conditions. Telehealth intervention holds considerable promise in addressing efficiently and effectively both caregiving challenges and the self-care concerns of family caregivers where and when assistance is needed. Therefore, clinicians should consider incorporating telehealth modalities for caregiver correspondence to assess emotional health and provide an avenue for caregivers to discuss support needs between scheduled healthcare visits.

## 5. Conclusion

The advancement and increased utilization of telehealth technologies offer unique opportunities to improve caregiver emotional functioning, health status, and caregiving skills. Findings of this review suggest that telehealth modalities are effective tools in delivering a variety of caregiver interventions, with significant improvements on several target caregiver outcomes across studies. Telehealth has been used most often to deliver skills training strategies to improve caregiver psychological well-being and enhance caregiver support. However, further research is needed to evaluate the efficacy of telehealth interventions in improving caregiving skills and caregiver self-efficacy, as well as caregiver health outcomes. Likewise, future studies need to examine the benefits of telehealth interventions with underserved, rural-residing caregiver populations.

## Figures and Tables

**Table 1:** Academic databases searched.

1. Ageline
2. AIDS and Cancer Research Abstracts
3. CINAHL
4. Cochrane Library
5. ComDisDome
6. Google Scholar
7. Gray Literature
8. Health and Safety Science Abstracts
9. MEDLINE
10. ProQuest Dissertations & Theses Full Text: Health & Medicine
11. PsycINFO
12. PubMed
13. Science Citation Index Expanded
14. Social Sciences Citation Index
15. Social Science Full Text
16. Social Services Abstracts
17. Web of Science

**Table 2:** Inclusion and exclusion criteria for article selection.

Studies included if:	Studies excluded if:
(1) Were telecommunication-based randomized controlled trials which focused on at least one caregiver outcome (i.e., health behaviors, psychosocial functioning, health service utilization, and cost or clinical outcomes) (2) Used at least one telehealth component in 50% of caregiver training sessions (e.g., service delivery over the Internet, telephone, videophone, point-to-point videoconference, and/or chat) (3) Were primarily focused on family caregiver intervention (may also include a care recipient training component) (4) The care recipient had a chronic medical condition (e.g., HIV/AIDs, progressive dementia, heart failure, psychiatric disabilities, and traumatic brain injury)	(1)Were written in languages other than English (2) Were duplicate articles (3) Were proposals or written descriptions of intervention programs, usability studies, quasi-experiments (i.e., used control groups, but not randomized), feasibility studies, or largely qualitative studies that lacked quantitative measures of caregiver health behavior or clinical outcomes (4) Used telehealth technologies solely to evaluate data on physical signs and symptoms of a condition, physiological indicators of health, and/or to provide emotional support (5) Focused exclusively on improvements in caregiver's knowledge and attitudes

**Table 3:** Telephone interventions for caregivers of persons with chronic health conditions.

Telephone interventions (*n* = 33)					
Authors	Telecommunication modalities employed	Sample/caregiver group	Study measures	Intervention description	Findings
Au et al. [13]^∗^	Telephone	Caregivers only Family caregivers of persons with dementia	(i) CESD (ii) Relationship Assessment Scale	Length: 5 months. Description: all participants received the same psychoeducational telephone-based intervention for the first 4 weeks, then randomized into 2 groups.Intervention: (*n* = 31) 8 biweekly telephone-based psychoeducation with behavioral activation sessions focused on pleasant event scheduling and improving communications. Comparison: (*n* = 31) 8 biweekly telephone-based sessions of general discussion of psychoeducation and related information.	Participants in the intervention group had decreased levels of depression versus the comparison group at endpoint. Relationship satisfaction also was significantly higher between time 2 and 3 in the intervention group; however, it did not improve significantly over time in the comparison group.

Bakas et al. [14]^∗^	Telephone	Caregivers only Older family caregivers of stroke survivors	(i) Revised Life Orientation Test (ii) Oberst Caregiving Burden Scale (difficulty subscale) (iii) Appraisal of Caregiving (threat subscale) (iv) PHQ Depression Scale (v) BCOS (vi) SF-36 (general health subscale)	Length: 8 weeks, with follow-up at 4 and 12 weeks.Intervention: (*n* = 26) mailed a booklet on stroke caregiver tips, stress management workbook, and an American Stroke Association (ASA) brochure; received 8 weekly calls from a nurse who assessed and addressed their current needs. Attention control: (*n* = 24) mailed ASA brochure. They received 8 weekly calls from a nurse who provided active listening and paraphrasing, referring any questions or concerns to healthcare provider or ASA.	Significant increases in caregiver self-reported optimism were noted at 4 weeks in the intervention group versus the comparison group and were maintained at the end of the intervention (8 weeks) as well as 12 weeks postintervention. Caregivers receiving the intervention also reported improved threat appraisal at 8 weeks as well as 12 weeks.

Bakas et al. [15]^∗^	Telephone	Caregivers only Stroke caregivers, mostly family	(i) PHQ-9 (ii) BCOS (iii) BRFSS (2 items)	Length: 8 weeks; 12-, 24-, and 52-week follow-up. Description: all participants received 8 weekly telephone sessions, with a booster at 12 weeks.Intervention: (*n* = 123) received a resource guide and an American Heart Association pamphlet, *Caring for Stroke Survivors*, in addition to 5 skill building tip sheets focusing on screening for depressive symptoms, maintaining realistic expectations, communicating with healthcare providers, problem-solving, strengthening existing relationships, and stress management. Telephone calls focused on training caregivers to identify and prioritize their needs and concerns and using innovative skill building strategies for management. Attention control: (*n* = 131) received only the American Heart Association pamphlet. Telephone calls focused on providing support using active listening strategies.	Among caregivers with depressive symptoms scores ≥5, those in the intervention group had a greater reduction in depressive symptoms from baseline to 8, 24, and 52 weeks and greater improvement in life changes from baseline to 12 weeks versus the comparison group. However, this did not hold for the total sample. Caregivers in the intervention group also had a greater reduction in unhealthy days from baseline to 8 weeks, yet this was not sustained at 12, 24, or 52 weeks.

Campbell et al. [16]	Telephone	Caregiver-care recipient dyads Partner-caregivers of African American men surviving prostate cancer	(i) Self-Efficacy for Symptom Control Inventory (partner version-adapted, activity efficacy, coping, symptom management subscales, plus total) (ii) Profile of Mood States	Length: 7 weeks.Intervention: (*n* = 20) weekly phone calls over 6 weeks focused on problem-solving, relaxation, communication, side effect management, cognitive restructuring, and maintenance planning. Usual care: (*n* = 20) standard outpatient medical care.	Caregivers reported no significant differences in anger, confusion, depression, fatigue, anxiety, or vigor. There were no significant effects for caregiver strain or self-efficacy between groups.

Chodosh et al. [17]	In-person visits at home and/or in the community plus telephone and mail versus telephone and mail only	Caregiver-care recipient dyads Informal caregivers of persons with dementia	(i) ZBI (ii) RMBPC (iii) PHQ-9 (iv) Caregiver Quality of Life Instrument (spirituality and faith; benefits of caregiving subscales) (v) Health Utilities Index	Length: 12 months; follow-up data collection at 6 & 12 months. Description: all participants received a minimum of 7 contacts from care managers (i.e., social workers) covering areas such as stress management techniques, problem-solving, educational needs, and advance care planning. Intervention: (*n* = 71 dyads) in person home visits (primarily), but also telephone and mail. Comparison group: (*n* = 73 dyads) telephone or mail contacts only.	In person dyads received, on average, one in-person contact during the study duration (*M* = 1.1, SD = 3.6) and more total telephone contacts than did the comparison group. Care quality improved substantially over time in both groups. Caregiver burden, care-recipient problem behaviors, and healthcare utilization did not differ across groups.

Davis et al. [18]^∗^	In-person, telephone	Caregiver-care recipient dyads Family caregivers of older adults with Alzheimer's-type dementia	(i) The Screen for Caregiver Burden (objective & subjective subscales) (ii) Geriatric Depression Rating Scale (iii) Interpersonal Support Evaluation List (iv) Life Satisfaction Index-*Z*	Length: 12 weeks with a 3-month follow-up. Intervention: (*n* = 24) 12 weekly telephone-based appraisal and coping skills training Comparison: (*n* = 23) 12 weekly home-based appraisal and coping skills training Attention control: (*n* = 23) weekly “friendly” supportive telephone calls only.	Method of intervention delivery affected outcomes and timing of improvements. Caregivers receiving the intervention in-person reported significant reductions in caregiver burden and distress at posttest and follow-up. Caregivers receiving the intervention by phone did not show statistically significant reductions at posttest but did show reductions at follow-up. Caregivers in the attention control reported significant reductions in burden and distress at posttest but this was not sustained at follow-up. There were no effects for caregiver depression, perceived social support, or life satisfaction.

Demiris et al. [19]	In-person, videophone	Caregivers only Older family caregivers of patients receiving in-home hospice care	(i) Caregiver Quality of Life Index-Revised (ii) The State-Trait Anxiety Inventory (iii) Problem-Solving Inventory	Length: 2 weeks. Description: received the intervention either in-person (*n* = 77) or by videophone (*n* = 49). The first session was conducted in-person for both groups, including consent, randomization, identification of 3 problems on checklist, and installation of videophone if applicable. For both groups, a total of three contacts were made between days 5 and 18 of hospice admission. Each contact lasted about 45 minutes. The sessions focused on problem-solving training.	Method of intervention delivery did not affect outcomes. Videophone-delivered intervention was not inferior to in-person intervention. Caregiver quality of life improved and state anxiety decreased under both conditions, and the observed changes in scores were similar for each group.

Dionne-Odom et al. [20]	Telephone	Caregivers only Family caregivers of persons with advanced cancer receiving palliative care	(i) CESD (ii) PG-13	Length: 3 weekly contacts after advanced cancer diagnosis, then monthly phone calls, including 1 after the death of the care recipient. Follow-up data collection at 6, 12, 18, and 24 weeks, then every 12 weeks until the care recipient expired. Intervention: (*n* = 19) participants received telephone intervention at the time of care recipient's diagnosis of advanced cancer and completed after-death questionnaires. Delayed/comparison: (*n* = 25) participants received delayed telephone intervention, beginning 12 weeks later, and completed after-death questionnaires.	Groups did not differ by early versus delayed intervention, on follow-up measures of depression and grief.

Dollinger and Chwalisz [21]^∗^	Telephone	Caregivers only Rural family caregivers of older adults	(i) Outcomes Questionnaire-45 (symptom distress, interpersonal relationship, and social role functioning subscales) (ii) Social Provisions Scale (guidance, reliable alliance, reassurance of worth, attachment, social integration, and opportunity for nurturance)	Length: 8 weeks, with 6-month follow-up. Intervention: (*n* = 42) received a manualized, but flexible intervention addressing knowledge, problem-solving skills, social support, and affect. Comparison: (*n* = 49) received access to a caregiver-initiated call-in helpline staffed by masters level counselors, with content drawn from the same manual, based on the presenting concern. Control: (*n* = 39) no intervention.	Over time, caregivers in the control group reported increased symptoms of distress, and increased difficulties in social roles and interpersonal relations. Caregivers in the comparison group also reported increased symptoms of distress and increased difficulties in interpersonal relations. Caregivers who completed the intervention reported significantly less stress, and improved social role functioning that was still evident at 6 months. Scores on the opportunity for nurturance subscale also decreased significantly over time in the intervention group.

Elliott et al. [22]^∗^	In-person (initial session for all groups), videoconferencing	Caregiver-care recipient dyads Family caregivers of persons with spinal cord injury	(i) SPSIR (ii) Inventory to Diagnose Depression (iii) SWLS	Length: 12 months; follow-up at 6 months and postintervention at 12 months. Description: both groups were asked to identify and prioritize problems unique to their situation. Intervention: (*n* = 29) individualized problem-solving training delivered via videoconferencing sessions that focused on (a) problem definition, (b) optimism and orientation toward problem-solving, (c) creativity and generating alternatives, (d) understanding and decision-making, and (e) solving the problem with implementation and evaluation of a solution. Attention control: (*n* = 32) weekly telephone contacts to discuss educational materials received by mail and monthly face-to-face interactions with the videoconferencing device.	There was a significant decrease in depression among caregivers in the intervention group at 6 months. Caregivers in the intervention group also were projected to improve social functioning over 12 months, while the comparison group was projected to decline. There were no statistically significant findings for caregiver satisfaction with life, quality of life, or problem-solving styles.

Elliott and Berry [23]^∗^	In-person (4 additional contacts in intervention group only), telephone	Caregiver-care recipient dyads Family caregivers of women with disabilities	(i) CES-D (ii) SWLS (iii) PILL (iv) SPSIR	Length: 48 weeks. Intervention: (*n* = 38) in-home problem-solving training sessions at months 1, 4, 8, and 12. Monthly telephone calls were conducted on the 8 alternate months. Attention control: (*n* = 43) monthly education-only telephone calls reviewing previously mailed health-education materials on topics such as emotions, relaxation, dental health, osteoporosis, exercise, respite, and long-term care.	Depression scores decreased over time in the intervention group, while depression scores for the comparison group increased. Constructive coping increased over time in the intervention group, while constructive coping decreased over time in the comparison group. No effects were observed for caregiver health or life satisfaction.

Farran et al. [24]	Telephone	Caregivers only Family caregivers of older adults with Alzheimer's or other dementia	(i) CES-D (ii) Behavior Management Scale-Revised (iii) Revised Memory and Problem Behavior Checklist	Length: 12 weekly sessions (5 weekly group and 7 weekly individual phone sessions), followed by group booster sessions at 6 and 12 months, and as-needed contacts throughout the 12-month intervention period. Intervention: (*n* = 141) caregiver skill building related to behavioral symptoms of dementia, as well as self-efficacy in managing those symptoms. The telephone calls were used to address individual needs. Comparison: (*n* = 154) standardized implementation of psychoeducation methods, as well as general information and support. These were not tailored to individual needs in phone calls.	The intervention and comparison groups did not differ in efficacy; there were significant improvements over time in caregiver mood, caregiver confidence in behavior management skills, and number of reported disruptive behaviors of the care recipient. The groups did not differ in time to institutionalization.

Farran et al. [25]^∗^	Telephone, in-person	Caregivers only Family caregivers of persons with dementia	(i) Subjective Caregiving Burden Scale (ii) CESD (iii) Positive and Negative Affect Scales (iv) CHAMPS	Length: 12 months, including a home visit at baseline, 6 and 12 months, and tapered telephone follow-up. Data were collected in-person at baseline, 6, and 12 months and via telephone at 3 and 9 months. Intervention: (*n* = 106) caregivers received the *Enhanced Physical Activity Intervention (EPAI)* via in-person and telephone to increase total and total moderate physical activity, plus caregiver skill building information. Intervention/comparison: (*n* = 105) caregivers received the *Caregiver Skill Building Intervention (CSBI)* in-person and via telephone only.	No significant mean differences between the EPAI and CSBI for any of the mental health variables at either baseline or 12 months were noted, but there were significant interactions between the EPAI and increased positive affect at both 6 and 12 months. The EPAI group participants increased physical activity over the course of the study, while he CSBI group tparticipants decreased physical activity over that period.

Finkel et al. [26]	Telephone (CTIS system), in-person (initial and last session for intervention only)	Caregivers only Older family caregivers of older adults with Alzheimer's or other dementia	(i) CES-D (ii) RMBC-24 (iii) Caregiver Health & Health Behaviors Scale (iv) Inventory of Socially Supportive Behaviors (received social support subscale)	Length: 24 months. Intervention: (*n* = 23) received a screen phone intervention providing information about the disease, community resources, and strategies to enhance safety, communication, self-care, social support, and management of problem behaviors. Teleconference calls included 8 individual educational and 6 group support sessions. Initial and last sessions were in-person. Comparison: (*n* = 23) received basic educational materials, two brief telephone check-in calls at three and five months postrandomization and were invited to participate in a workshop following the 6-month assessment.	No significant main effects for treatment were noted. Two significant interactions were found: caregivers with higher baseline depression scores who received the intervention had significantly greater reductions in posttest depression scores. Similarly, among caregivers at baseline who reported a higher level of support, those provided the intervention were more likely to report receiving similar levels of support by posttest, compared to reduced support received by the comparison group participants.

Gant et al. [27]	Telephone, video modules (intervention group only; mailed to client, format not listed in article)	Caregivers only Male caregivers of a family member with dementia	(i) Researcher-Developed Likert Scale Items of How “Bothered or Upset” or “Irritated or Annoyed” the Caregiver Became When Behavioral Problems Occurred (ii) The Revised Scale of Caregiving Self-Efficacy (iii) Positive and Negative Affect Scale (iv) Interviews	Length: 12 weeks. Intervention: (*n* = 17) a 10-session video series, caregiving workbook, and 12 weekly phone calls. The intervention was behaviorally focused, utilizing videos (VHS) along with homework and discussions focused on each participant's unique problems. Comparison: (*n* = 15) 7 biweekly phone calls to review materials, discuss use of strategies, and answer any questions using a standardized script.	Participants in both conditions improved significantly, with no evidence of greater efficacy in the intervention group for reducing psychosocial distress, increasing positive affect, or caregiving self-efficacy, compared to the more intense and more structured intervention.

Grant et al. [28]^∗^	Telephone, in-person (initial session for intervention group only)	Caregivers only Older family caregivers of stroke patients recently discharged from inpatient rehabilitation	(i) SF-36 (ii) SRSIR (iii) The Client Satisfaction Questionnaire (iv) CES-D (v) Preparedness for Caregiving Scale (vi) Caregiving Burden Scale (difficulty subscale)	Length: 12 weeks. Description: 74 caregivers were randomized to 1 of 3 groups. Intervention: an initial in-home session within a week of discharge from the hospital, followed by weekly phone calls at week 2, 3, 4, and biweekly calls at weeks 6, 8, 10, and 12 postdischarge. The problem-solving intervention followed the same general structure for each of the top 3 caregiver concerns at the point of contact: identifying problems, listing solutions, choosing and testing a solution, and evaluating outcomes. Attention control: telephone calls (same timing), but caregivers were asked to report specific data on a healthcare utilization since the last contact. Control: usual care.	The intervention group showed relatively greater improved scores in vitality, role limitations, mental health, negative problem orientation, rational problem-solving, impulsivity-carelessness, caregiver preparedness, and caregiver depression.

Hartke and King [29]^∗^	Telephone, in-person (only some intervention group members)	Caregivers only Spouse caregivers of stroke survivors after rehabilitation	(i) CES-D (ii) UCLA Loneliness Scale (iii) Caregiver Competence Scale (iv) The Burden Interview (v) The Pressing Problem Index	Length: 8 weeks, with a 6-month follow-up. Intervention: (*n* = 68) structured, psychoeducational training based on stress and coping theory was delivered via 8 one-hour small-group telephone-based sessions. A group stress management manual was mailed, as well as a list of contact information for group members. Comparison: (*n* = 55) received the stress management manual and a brief written description of caregiver stress and stroke.	The comparison group showed a significant increase in burden during the study; intervention group showed a significant increase in self-rated competence.

Hu et al. [30]^∗^	Telephone	Caregivers only Family caregivers of persons with heart failure	(i) ZBI (ii) SF-36 (physical component summary & mental component summary) (iii) CES-D	Length: 3 months; plus a 3-month postintervention follow-up. Intervention: (*n* = 51) multidisciplinary education and support group meetings in person, plus weekly nurse telephone calls for 3 months and as needed telephone contacts. Cultural sensitivity was included for the Chinese culture, including effects of role changes and available resources. Usual care: (*n* = 51) standard care only.	There were no significant differences at baseline in outcome variables. At posttest and 3-month follow-up, the experimental group reported significantly less burden, greater QoL related to mental health, and less depression. No differences were found in QoL related to physical health.

Keeping-Burke et al. [31]^∗^	Telephone, videoconferencing, patient vital signs monitoring system equipment	Caregiver-care recipient dyads Caregivers of patients undergoing elective, first-time coronary artery bypass graft surgery	(i) State-Trait Anxiety Inventory (anxiety subscale) (ii) CES-D	Length: 1 week; 3-week follow-up. Intervention: (*n* = 91; dyads) standard care, plus follow-up audio-visual telehealth sessions for heart monitoring, symptom reports and discussion of symptoms, and review of postoperative care instructions. Usual care: (*n* = 91 dyads) standard care only, which included education prior to the surgery, daily education and physical therapy during hospitalization, and a scheduled follow-up with the surgeon in six weeks.	Caregivers' levels of depressive symptoms decreased significantly more for those in the intervention group, compared to control. Caregivers of male patients in the intervention group experienced a greater decrease in anxiety from presurgery to 3 weeks after discharge than did caregivers of male patients who were in the control group. There was no evidence that the intervention had a greater impact than control on patient anxiety from presurgery to 3 weeks after discharge. Patients who received the intervention were less likely than the control to have contacted a physician during the first 3 weeks after hospital discharge postsurgery.

King et al. [32]^∗^	Telephone, in-person (initial in-home session)	Caregivers only Older female family caregivers of persons with dementia	(i) Short Form General Health Survey Screen for Caregiver Burden (ii) CHAMPS (iii) Cardiovascular Reactivity to Emotional Challenge (iv) Pittsburgh Sleep Quality Index (v) PSS (vi) BDI (vii) Block95 Food Frequency Questionnaire	Length: 12 months. Intervention: (*n* = 51) one at-home session to set up an exercise program, then follow-up telephone contacts were conducted biweekly for the first 2 months, then once monthly through the last 10 months for a total of 14 contacts. Contacts lasted 15-20 minutes to monitor exercise progress, answer questions, and provide feedback. Participants completed and mailed daily logs to the study team monthly. Comparison: (*n* = 49) one at-home session to describe the nutritional program then telephone contacts to follow up. Telephone contacts followed the same schedule as the intervention. Phone contacts focused on different topics each month. Participants completed and mailed daily logs to the study team monthly.	The intervention group had significantly greater increase in self-reported activity level and sleep quality, as well as reduced systolic and diastolic blood pressure reactivity, compared to the comparison group. Conversely, the comparison group reported significantly greater reduction in consumption of high-fat and high-sugar foods, and in fat as a percentage of total calories. Both groups reported reduction in psychological distress and depression, with no significant difference between the groups.

Kwok et al. [33]^∗^	DVD with information, telephone	Caregivers only Family caregivers of persons with dementia of any stage	Chinese Versions (i) ZBI (ii) Caregiving Self-Efficacy-Revised	Length: 12 weeks. Intervention: (*n* = 20) an educational DVD, plus a psychoeducation program delivered over the telephone in 12 weekly sessions. Topics included information about dementia, skills of communicating with the patient, management of behavioral and psychological symptoms of dementia, caregivers' own emotional issues, resources available in the community, and long-term care plan. Comparison: (*n* = 22) DVD of educational information about dementia caregiving only.	Caregivers receiving the intervention obtained significantly larger change scores on burden and on caregiving self-efficacy-obtaining respite.

Lingler et al. [34]	In-person, telephone	Caregiver-care recipient dyads Informal family caregivers who had difficulty managing medications for persons with memory loss	(i) MedMaIDE (ii) Medication Deficiency Checklist (Researcher-Developed)	Length: 16 weeks (8 weeks active intervention, 8 weeks maintenance/check-in). Intervention: (*n* = 42) participants received a booklet covering 7 key areas of medication management. A nurse or social worker reviewed these areas with the caregiver and provided problem-solving support in 2-3 home visits over 4-6 weeks followed by 2-3 telephone sessions over 2-4 weeks. Skills were reinforced through 4 biweekly telephone calls over 8 weeks. Control: (*n* = 41) participants received a resource manual and medication reconciliation in-person at baseline, plus the intervention manual at the completion of the study.	Both groups showed significant decreases in medication management problems at follow-up.

McCann et al. [35]^∗^	In-person, telephone	Caregivers only Family caregivers of persons with moderate depression receiving outpatient treatment	(i) ECI	Length: 3 months (8 weeks intervention; 1-month follow-up). Intervention: (*n* = 27) participants received a cognitive behavioral-based, guided self-help manual geared towards individuals with depression xand/or their caregivers and were asked to complete one module per week for 8 weeks. Weekly follow-up brief telephone calls were conducted for 8 weeks to answer questions and provide support. Also received occasional unplanned in-person visits at outpatient medical appointments. Attention control: (*n* = 27) participants received brief telephone calls on a similar schedule to respond to questions and provide brief support. Occasional unplanned in-person visits at outpatient medical appointments were also provided.	Caregivers showed a significant decrease in negative experience and a significant increase in positive experience postintervention, which was maintained at the one-month follow-up.

Porter et al. [36]	Telephone	Caregiver-care recipient dyads Family caregivers of persons with lung cancer (stage I-III) at any point in treatment	(i) Profile of Mood States-B (ii) CSI (iii) Self-Efficacy Scale (modified)	Length: 8 months, with 4-month follow-up. Description: dyads received equal number and length of sessions. Dyads participated together via speakerphone in all sessions. Intervention: (*n* = 117 dyads) received coping skills training which included symptom management and stress management strategies, with caregivers being encouraged to adopt a “coach” role, and to utilize coping for self-care. Comparison: (*n* = 116 dyads) received cancer education and support tailored to the stage and treatment of each patient, such as lung cancer symptoms and treatments, as well as information about palliative versus curative care.	No significant differences in outcomes between the intervention and the comparison group were noted. There was a significant effect of time: caregivers in both groups reported increased self-efficacy and reduction in anxiety over time. Caregivers of patients with less advanced cancer in the comparison group had significant reduction in strain and increased self-efficacy, whereas those caring for patients with more advanced cancer responded better to the intervention.

Rivera et al. [37]^∗^	Telephone, 4 additional in-home visits for intervention group only	Caregiver-care recipient dyads Family caregivers living with adult TBI survivors	(i) CES-D (ii) SWLS (iii) PILL (iv) Caregiver Burden Scale (difficulty subscale) (v) SPSIR	Length: 12 months. Intervention: (*n* = 33) problem-solving training including 4 in-home sessions teaching problem-solving skills and 8 telephone calls, spread across 1 year. The intervention was tailored to individuals' identified problems and priorities. Attention control: (*n* = 34) received educational materials in the mail, plus monthly follow-up calls to discuss the information; these telephone calls were 10-15 minutes in length, on average.	The intervention group reported significantly greater reduction in depression, health complaints, and “dysfunctional problem-solving”, compared to the comparison group. Results on other outcome measures were not significant.

Shaw et al. [38]	Telephone	Caregivers only Caregivers and friends of persons diagnosed with poor prognosis upper gastrointestinal or Duke D colorectal cancer	(i) SF-12 (ii) Caregiver Reaction Assessment Scale (iii) Distress Thermometer (1-item) (iv) Supportive Care Needs Survey-Caregiver (v) Health Services Utilization Index	Length: 10 weeks posthospital discharge, with follow-up at 3 and 6 months. Intervention: (*n* = 64) participants received a standardized manual providing education related to domains of patient care, family relationships, and emotional and physical care. Individualized education and support were provided in 4 standardized telephone calls over 10 weeks posthospital discharge by clinical psychologists. Usual care: (*n* = 64) standard care only.	At the 3-month and 6-month posthospital discharge intervals, no significant differences were noted between the intervention and usual care participants across all caregiver measures. However, intervention participants reported significantly fewer patient emergency department visits and unplanned hospital readmissions at the 3-month postdischarge, but this difference was not maintained at the 6-month postdischarge interval.

Smith and Toseland [39]^∗^	Telephone	Caregivers only Spouse and adult child caregivers of frail older adults with significant need for assistance with activities of daily living	(i) MOS Social Support Survey (ii) ZBI (iii) CES-D (iv) State-Trait Anxiety Inventory (v) Pressing Problems Index (vi) Community Services Inventory	Length: 12 weeks. Intervention: (*n* = 53) telephone-based therapy delivered to small groups of spouse or adult children caregivers, led by a social worker who guided them through strategies for emotion-focused coping (stress inoculation training), problem-focused coping, and use of group/peer support. Usual care: (*n* = 44) standard care, offered by a senior services center to which all were referred, with opportunity to participate in a telephone support group after 12 weeks and completion of the follow-up assessments.	No significant differences between groups were noted in spouses on any of the outcome measures. Positive social interaction and emotional/informational support increased for adult children in the intervention group but decreased for those in the control group. Adult children in the intervention group had significant decreases in total strain over time, while adult children in the control group had increases in total strain. Depressive symptoms decreased significantly more in adult children in the intervention group versus the control. There were no significant findings for anxiety. Decreases in the level of stress of pressing problems over time were significantly greater in adult children in the intervention group than those in the control group. Adult children in the intervention group also reported significantly greater feelings of effectiveness to manage pressing problems over time, compared to those in the control group. Adult children in the intervention group had increased knowledge of services, greater knowledge of how to access services, and used more services over time, whereas adult children in the control group showed decreases in all three of these measures.

Tanner et al. [40]	In-person, telephone	Caregiver-care recipient dyads Informal caregivers of elders with a memory disorder living at home	(i) JHDCNA (ii) ZBI (iii) Geriatric Depression Scale (iv) Researcher-Developed Items on Caregiver Burden & Perceived Daily Difficulty Providing Care (V) Self-Rated Overall Health (Vi) Self-Rated Overall Stress	Length: 18 months; data collected at baseline, 9 months, & 18 months. Intervention: (*n* = 106 dyads) 18 months of care coordination by an interdisciplinary intervention team that included nonclinical community workers (coordinators) as the frontline staff, a registered nurse, and a geriatric psychiatrist. The manualized care coordination protocol consisted of four key components: (1) identification of needs and individualized care planning based on the JHDCNA to address unmet needs and to match the priorities and preferences of the dyad and family; (2) provision of dementia education and skill building strategies; (3) coordination, referral, and linkage to health and community services; and (4) care monitoring. There were a minimum of 2 in-home visits and monthly telephone contacts provided, with opportunity for more home-visits, visits at medical appointments, and additional telephone contacts (additional contacts not tracked). Comparison: (*n* = 183 dyads) received the written needs assessment results and intervention recommendations, as well as a brief resource guide developed for the study that provided program and contact information for local and national aging service organizations.	There were no statistically significant group differences in reduction of total percent of unmet needs from baseline to 18 months or in any of the four need domains; however, the total percent of unmet needs decreased in both the comparison and intervention groups when modeled independently using mixed effects linear regression models. There was a decrease in hours per week caregivers spent with care recipients in the intervention group relative to the comparison group from baseline to 18 months and an increase in the control group, but this was not statistically significant after multiple comparison correction. Similarly, though not statistically significant, ZBI score estimates increased in the comparison group and decreased in the intervention group.

Tremont et al. [41]^∗^	Telephone	Caregivers only Caregivers of person with dementia	(i) ZBI (ii) Geriatric Depression Scale (iii) RMBPC (iv) Alzheimer's Disease Knowledge Test (v) SF-36 General Health (vi) Self-Efficacy Scale (vii) Family Assessment Device (viii) Multidimensional Scale of Perceived Support	Length: 52 weeks. Description: all participants received an informational binder with local resources and educational materials from the Alzheimer's Association. Intervention: (*n* = 32) a multicomponent, manualized program delivered by telephone, providing emotional support, directing caregivers to appropriate resources, encouraging caregivers to attend to their own physical, emotional, and social needs, and teaching caregivers strategies to cope with ongoing problems. Usual care: (*n* = 28) standard care provided.	Caregivers who completed the intervention reported significantly lower perceived burden compared to caregivers in the control group. Intervention caregivers also reported significantly less severe reactions to memory and behavior problems, but there was no significant difference between the groups in reported depression. No other outcomes were significant.

Tremont et al. [42]^∗^	Telephone	Caregivers only Family caregivers of persons with dementia	(i) CES-D (ii) ZBI (iii) RMBPC (iv) Family Assessment Device (v) Self-Efficacy Questionnaire (vi) Positive Aspects of Caregiving Scale (vii) Euro QOL	Length: 6 months; data collected at baseline and 6 months. Intervention: (*n* = 133) family intervention telephone tracking-caregiver: 6 telephone contacts distributed over 6 months that focused on providing dementia education, emotional support, directing caregivers to appropriate resources, encouraging caregivers to attend to their physical, emotional, and social needs, and teaching caregivers strategies to cope with ongoing problems. Comparison: (*n* = 117) telephone contacts to provide supportive counseling only with identical frequency/timing of contacts. Content and process differed substantially.	The intervention group had significantly improved caregiver depressive symptoms and less severe reactions to care-recipient depressive behaviors compared with the comparison group.

Wilz et al. [43]^∗^	In-person (initial visit for both intervention and comparison groups), telephone	Caregivers only Family caregivers of older adults with dementia	(i) Goal Attainment Scaling (ii) Researcher-Developed Questions regarding Satisfaction with Various Intervention Elements and Perceived Benefit	Length: 3 months, with 6 months postintervention follow-up. Intervention: (*n* = 126) a telephone-based cognitive-behavioral therapy intervention with modules focused on utilization of social and professional support, problem-solving and coping with problem behavior of the patient, modifying dysfunctional thoughts, and expressing and processing emotions of role change and loss. Goal setting and attainment was included. Program consisted of 7 (60 minutes) therapeutic sessions; the first 4 sessions took place on a weekly basis, sessions 5-6 on a fortnightly basis, and session 7 monthly. Comparison: (*n* = 53) A telephone intervention with psychologists who provided an equal number of training sessions in progressive muscle relaxation (PMR) for managing distress and anxiety associated with caregiving. Participants were also provided a CD program to follow. Usual care: (*n* = 50) treatment as usual, including written educational material about dementia and dementia caregiving with the addresses of local self-help organizations that was dispersed to all groups.	Caregivers in the intervention group reported significant progress toward personally identified goals. Both groups reported high satisfaction and benefit of the interventions, but the intervention group rated the intervention even more helpful, reported a higher amount of fulfilled expectations, showed a higher percentage of willingness to recommend the intervention, and expressed a more intense desire to participate in such a trial again than the comparison group.

Wilz and Soellner [44]^∗^	In-person (initial session for both intervention and comparison group), telephone	Caregivers only Family caregivers of persons with dementia	(i) CES-D (German) (ii) Gießener Beschwerde-Bogen-24 (iii) Visual Analog Scales	Length: 3 months; data collected at baseline (prerandomization), 3- & 6-month postintervention. Intervention: (*n* = 91) educative and resource materials plus seven 1-hour therapeutic telephone sessions; the first session in-person, sessions 2-4 weekly, sessions 5-6 every 2 weeks, and session 7 a month later. Treatment control: (*n* = 36) educative and resource materials plus training in progressive muscle relaxation in addition to CBT. First session in-person at caregiver's home, with 6 subsequent phone sessions at same frequency as intervention. Usual care: (*n* = 39) educative and resource materials only.	The intervention group showed short-term effects in improved well-being compared with the treatment control and the untreated control groups. In comparison to the untreated controls, the intervention was effective in decreasing body complaints at posttreatment and in improving perceived health at a 6-month follow-up. The intervention group also showed improvements in depressive symptoms at a 6-month follow-up as compared to the treatment control group.

Winter and Gitlin [45]	Telephone	Caregivers only White and African American female family caregivers of persons with Alzheimer's and related dementias	(i) CES-D (ii) ZBI (iii) Kaye's Gain Through Group Involvement Scale	Length: 6 months. Intervention: (*n* = 58) a telephone support group intervention based upon stress process theory, providing emotional support and validation, and promoting mutual coping assistance among the participants. Usual care: (*n* = 45) standard care.	No large or statistically significant differences were noted between the intervention and control groups at 6 months on the outcome measures. There were no significant main effects of treatment or treatment intensity (# of sessions attended), but there was a significant interaction effect showing that older caregivers receiving the intervention reported lower depression at 6 months than those in the control group (although depression scores were higher compared to baseline, for both groups). Number of sessions attended was not associated with depression, caregiver burden, or perceived gains at 6 months. More frequent session attendance was predicted by younger age, spousal (wife) relationship, and being African American.

^∗^Study reported significant improvement in at least one target outcome in the intervention group as compared to the control/comparison group. CESD: Center for Epidemiological Studies-Depression Scale; PHQ: Patient Health Questionnaire; BCOS: Bakas Caregiving Outcomes Scale; BRFSS: Behavioral Risk Factor Surveillance System; RMBPC: Revised Memory and Behavioral Problem Checklist; ZBI: Zarit Burden Interview; PG-13: Prigerson Inventory of Complicated Grief-Short Form; CHAMPS: Community Health Activities Model Program for Seniors; SF-36: Short Form 36; CSI: Caregiver Strain Index; MedMaIDE: Medication Management Instrument for Deficiencies in the Elderly; ECI: Experience of Caregiving Inventory; CPMQ: Caregiver Pain Medicine Questionnaire; TBI: traumatic brain injury; PART-O: Participation Assessment with Recombined Tools-Objective; SF-12: Short Form 12; JHDCNA: Johns Hopkins Dementia Care Needs Assessment; SPSIR: Social Problem-Solving Inventory-Revised; SWLS: Satisfaction with Life Scale; PSS: Perceived Stress Scale; MOS: Medical Outcomes Study; PILL: Pennebaker Inventory of Limbic Languidness Scale.

**Table 4:** Web interventions for caregivers of persons with chronic health conditions.

Web interventions (*n* = 14)					
Authors		Caregiver group	Study measures	Intervention description	Findings
Beauchamp et al. [46]^∗^	Asynchronous web-based modules with tailoring and videos	Caregivers only Caregivers of family members with dementia who maintained outside employment	(i) CES-D (ii) State-Trait Anxiety Inventory (iii) Caregiver Strain Scale (iv) Positive Aspects of Caregiving	Length: 4 weeks. Intervention: (*n* = 150) access to asynchronous web-based modules and videos (caregiver's friend: dealing with dementia) and educational materials tailored to care recipient and care giver characteristics. The coping strategies presented throughout focused on problem-focused techniques and social support skills. Wait list control: (*n* = 149) usual treatment, then given access to the intervention.	Those in the intervention group reported significant improvements in depression, anxiety, level and frequency of stress, caregiver strain, self-efficacy, intention to seek help, and positive aspects of caregiving.

Blom et al. [47]^∗^	Asynchronous web-based educational modules with email	Caregivers only Family caregivers of persons with dementia	(i) CESD (ii) HADS (iii) Informal Care Scale (self-perceived pressure subscale) (iv) RMBPC (v) Short Sense of Competence Questionnaire (vi) Pearlin Mastery Scale	Length: self-paced; 5-6 months from baseline with midpoint assessment at 3 months. Intervention: (*n* = 149) eight self-paced interactive web-based modules (*Mastery over Dementia*) which included problem-solving, relaxation, arranging help from others, cognitive restructuring, and assertiveness training, plus a booster summary session were delivered. After each session, participants completed and submitted homework to a coach who returned electronic feedback. Comparison: (*n* = 96) E-bulletins (digital newsletters) with practical information on providing care for someone with dementia were sent by email every 3 weeks over nearly 6 months. There was no contact with a coach.	Depression and anxiety improved significantly in the intervention group. Noteworthy, even older caregivers (>65 years) can benefit from a web-based intervention to reduce psychological symptoms.

Chih et al. [48]^∗^	Asynchronous web-based educational materials, interactive electronic patient reported outcomes, medical online platform which generated emails to clinicians in certain situations	Caregiver-care recipient dyads Family caregivers of persons with advanced stage lung, breast, and prostate cancer in outpatient clinics	(i) Family Care Inventory (caregiver preparedness subscale) (ii) Caregiver Burden Inventory (caregiver physical burden subscale) (iii) Shortened Version of the Profile of Mood States (negative mood items)	Length: 52 weeks, with 6- and 12-month follow-up. Intervention: (*n* = 118 dyads) asynchronous access to an interactive medical online platform with a wide variety of practical, cancer-specific educational and self-help coaching resources. Also given rating scales to report physical and psychological symptoms of caregiver and patient. Clinical reports of those symptoms were generated and made available to their medical providers, including an email alert when reported symptom intensity crossed a threshold. Comparison: (*n* = 117 dyads) access to the interactive website, but this did not generate a clinical report to their clinical team.	Caregiver burden and subjective preparedness did not differ between groups at 6- and 12-month postintervention, but caregiver reports of negative mood were lower for those in the intervention group.

Hattink et al. [49]^∗^	Web-based training modules plus online peer and expert communities for support and information exchange	Caregivers only Informal caregivers of persons with dementia; volunteers in dementia care; professional caregivers	(i) Alzheimer's Disease Knowledge Scale (ii) Alzheimer's Disease Survey (iii) Approaches to Dementia Questionnaire (iv) Interpersonal Reactivity Index (v) 3 Researcher-Developed Items (vi) Short Sense of Competence Questionnaire	Length: 2 to 4 months. Intervention: (*n* = 37) participants were given access to web-based interactive training modules plus online peer support and expert communities for information exchange. Wait list control: (*n* = 46) participants were given access to the training modules, online support, and expert communities after 4 months.	Lay people in both the intervention and control conditions showed positive change over time in reporting a person-centered approach to dementia care. No other measures of knowledge about and attitudes toward dementia were statistically significant across groups among laypeople or professionals. In pre-postcomparisons, participants in the intervention group reported less distress in tense situations, more empathy and concern for the well-being of other people and feeling better able to understand situations and the actions of other people versus the waitlist/control. However, participants in the intervention group reported feeling less competent in caregiving after the intervention.

Kajiyama et al. [50]^∗^	Asynchronous (educational modules with videos available online and on DVD). Workbooks also available and encouraged	Caregivers only Family caregivers of older adults with dementia	(i) PSS (ii) RMBPC (iii) CES-D (vi) Perceived Quality of Life	Length: 12 weeks. Intervention: (*n* = 75) asynchronous access to an online or DVD intervention program which included 6 skill building modules with video illustrations, as well as modules focused on reducing stress and negative thoughts designed to be completed in set order. They also received a workbook and were encouraged to fill it out. Comparison: (*n* = 75) received access to a website containing similar navigational features, with information about dementia as well as links to video-taped information plus booklet materials from various health agencies.	Perceived stress decreased significantly over time in the intervention group, whereas there was no significant change in perceived stress in the comparison group. The groups did not differ in the amount of change over time in feeling “bothered” by care recipients' memory deficits and behavior problems, nor did they differ in amount of change in depression or perceived quality of life.

Klemm et al. [51]^∗^	Asynchronous web-based	Caregivers only Mostly employed (part- or full-time) adults (≥40) caregivers of persons with a chronic disease, with 9 retired or full-time caregivers as well	(i) CES-D (ii) Modified CSI (iii) CQoL-I	Length: 12 weeks. Professionally facilitated intervention group: (*n* = 20) a psychiatric nurse provided psychoeducation through weekly posts on topics related to caregiving and responded to questions as requested. Moderated intervention: (*n* = 27) primarily peer-directed, this group was moderated by the primary investigator and utilized an unstructured format once initial guidelines and purpose had been posted. Usual care: (*n* = 39) standard care. No access to website and received no treatment.	The two intervention groups showed significant improvements in depressive symptoms and quality life as compared to the control group; the two intervention groups did not differ significantly on depressive symptoms or quality of life. There were no significant differences between groups for caregiver strain. Results suggest providing support to caregivers, regardless of format, can help improve quality of life and decrease depressive symptoms.

Marziali and Donahue [52]^∗^	Asynchronous website, synchronous web-based videoconferencing, email	Caregivers only Family caregivers of older adults with neurodegenerative diseases	(i) Composite of the HSQ-12 and the MOS-36 (ii) CES-D (iii) Caregiver Report: Activities of Daily Living and Instrumental Activities of Daily Living Performed for Care Recipient, Plus Stress Rating for Each (vi) RMBPC (v) Multidimensional Scale of Perceived Social Support	Length: 10 weeks. Intervention: (*n* = 33) asynchronous access to a website with information, email, and threaded discussion; a point-to-point video-conferencing link supported caregivers' participation in a manual-guided psychosocial support group for 10 weekly sessions, followed by an additional two sessions led by a group member. Usual care: (*n* = 33) standard care. No-intervention condition.	The intervention group showed reliable adherence to the manual-guided support group and similar themes were noted as compared to face-to-face support groups. The intervention group also reported significantly less stress postintervention, while the control group reported an increase in stress.

Petranovich et al. [53]^∗^	Synchronous web-based, in-person (initial visit for intervention group only)	Caregivers only Primary family caregivers of adolescents (12-17 years old) with mild to severe TBI within prior 1-7 months	(i) SCL-90-R (ii) CES-D (iii) CSES	Length: 6 months; follow-up at 6-, 12-, and 18-month postbaseline. Intervention: (*n* = 65) an initial 2-day intervention training program was provided to participating therapists. Family caregiver participants received an initial 1-day in-home training session to introduce the family intervention which focused on problem-solving training, communication skills, self-regulation, and anger management and to identify family goals to be addressed in the intervention. Participants received a detailed manual and completed a series of online modules and Skype sessions with a licensed therapist. Up to 4 supplemental modules were available for use as determined by the therapist. Comparison: (*n* = 67) a self-guided, web-based program which provided information about TBI and online resources. Participants were asked to access this 1 hour per week.	The intervention was effective in reducing parent distress but had minimal effect on depression and self-efficacy. The intervention was more effective in reducing distress in low-income families at 12- and 18-month follow-up than the control group. While depression was greater for caregivers of adolescents with severe TBI versus adolescents with moderate TBI, these differences did not continue through the 12- and 18-month follow-up.

Pierce et al. [54]	Asynchronous web-based education materials, email	Caregivers only Family caregivers of adult first-time stroke survivors discharged from rehabilitation center; novice Internet users	(i) CES-D (ii) SWLS (iii) Functional Independence Measure (iv) Healthcare Usage-Researcher Developed	Length: 52 weeks. Intervention: (*n* = 51) asynchronous access to web-based educational materials, customized “tip sheets”, email forum to ask questions of a multidisciplinary team (e.g., nurses, therapists, social worker, physician), and a nonstructured, nurse-facilitated email forum among all participants. Control: (*n* = 52) received usual medical care and were asked not to use the Internet for the year of the study.	No differences were found in reported depression and life satisfaction between the intervention and usual care groups. Significant differences were found between the groups on survivors' visits to hospital emergency departments (33% fewer visits for the intervention group) and number of hospital readmissions (66% fewer readmissions for the intervention group). No differences were found in number of visits to their healthcare providers.

Raj et al. [55]	In-person (initial visit intervention group only), synchronous web-based videoconferencing, asynchronous web-based educational modules	Caregivers only Parents/legal guardians of children with TBI	(i) Symptom Checklist-90-Revised (Global Severity Index) (ii) CES-D (iii) Parenting Stress Index (3^rd^) (iv) CSES	Length: 4-6 months, no follow-up. Intervention: (*n* = 20) participants received an initial in-home visit to introduce the intervention designed to increase positive parenting skills and improve caregiver stress management. Participants also received 10 core web-based sessions and up to 4 supplemental sessions. Each session included self-guided web content, followed by a videoconference call with a therapist to discuss content and practice parenting skills with live feedback. Comparison: (*n* = 20) participants received links to TBI web resources.	Parent income moderated treatment effects on parent psychological distress. Specifically, lower-income parents in the parenting skills group reported significant reductions in psychological distress compared with lower-income parents in the control group. No differences for caregiver depression, parenting stress and caregiver efficacy between the two groups over time.

Smith et al. [56]^∗^	Synchronous (audio only, Internet-based support groups) & asynchronous options (online library/educational videos, message boards, email)	Caregiver-care recipient dyads Wives caring for husbands surviving stroke	(i) CES-D (ii) Mastery Scale (9 items) (iii) Self-Esteem Scale (iv) MOS Social Support Survey-11 items	Length: 11 weeks, with 1-month follow-up. Intervention: (*n* = 19 dyads) asynchronous access to educational videos and an online library of educational materials related to stroke, caregiving, and coping. Participants took part in guided online synchronous audio-only chat sessions with other wives in the study twice weekly for a total of 17 sessions, and an email message board enabling messages exchanged with the nurse facilitator and with each other. Comparison: (*n* = 19 dyads) asynchronous access to an online educational resource library and completed measures at baseline and at two follow-up intervals.	Controlling for baseline depression, caregivers in the intervention group reported significantly lower depression one-month postintervention as compared to the control group. No significant treatment effects were noted for other outcomes.

Vander Stoep et al. [57]^∗^	In-person, synchronous web-based videoconferencing	Caregiver-care recipient dyads Caregivers of a child with ADH	(i) Parenting Strain Index (role restriction, isolation, and spouse subscales) (ii) PHQ-9 (iii) Caregiver Strain Questionnaire	Length: 25 weeks; parenting stress was measured at baseline & 25 weeks; all other domains were measured at baseline, 4, 10, 19, & 25 weeks. Intervention: (*n* = 111) children's ADHD Telemental Health Treatment Study-hybrid telehealth service-delivery model with combined pharmacotherapy and caregiver behavior training for reducing children's ADHD-related symptoms and caregivers' distress. Participants received 6 videoconferencing sessions led by a psychiatrist on pharmacotherapy, immediately followed by 6 in-person sessions of caregiver behavior training from master's level therapists spaced 3 to 4 weeks apart. Comparison: (*n* = 112) received primary care with a single teleconsultation session.	Overtime, caregivers in both service models reported significantly decreased levels of distress and depression symptoms and increased levels of family empowerment. Effects were significantly greater for caregivers in the intervention group. Further, combined child symptom improvements mediated reductions in parenting stress and caregiver strain, and improvements in caregiver strain were significantly mediated by treatment-induced decreases in child ODD symptoms.

Wade et al. [58]^∗^	Synchronous web-based videoconferencing and asynchronous educational modules with tailoring and videos	Caregiver-care recipient dyads Families of children (ages 5-16) with moderate to severe TBI between 1 and 24 months previously.	(i) Social Problem-Solving Index-Short (ii) CES-D (iii) Anxiety Inventory (iv) Symptom Checklist-90 (v) Global Severity Index	Length: 16-24 weeks. Intervention: (*n* = 26) manualized problem-solving therapy and cognitive behavioral skills training through synchronous online videoconferencing sessions, plus one in-person in-home initial interview. During the initial meeting, the therapist conducted a structured interview and elicited goals identified by caregivers. Subsequent meetings were conducted via synchronous online videoconferencing, and in-between times, the family completed web-based self-guided materials. There were 8 core sessions, with 4 additional supplemental sessions. Participants also received Internet access to a home page linking them to brain injury web sites and resources. Comparison: (*n* = 20) usual psychosocial care, plus access to the home page and links.	The intervention group reported significantly less depression, anxiety, and global distress at follow-up than did the control group, after controlling for baseline symptom levels. No significant findings were noted for problem-solving.

Wade et al. [59]	Synchronous web-based videoconferencing, asynchronous web-based educational modules, in-person (initial visit intervention group only)	Caregiver-care recipient dyads Family caregivers of adolescents (ages 11-18) with moderate to severe TBI between 3 and 19 months previously	(i) Social Problem-Solving Index (ii) CES-D (iii) Symptom Checklist-90 Global Severity Index	Length: 6 months. Intervention: (*n* = 21) web-based interactive teaching modules for 10 core topics, with up to six supplemental modules. Each module was followed by a point-to-point videoconference session to practice the module skills together. Structured materials included problem-solving strategies and TBI information, as well as individualized materials for needs such as pain management. Comparison: (*n* = 20) the same technology resources and online TBI-specific educational modules but no problem-solving modules or interactive video sessions; they were encouraged to spend one hour/week reviewing the informational websites.	Both groups improved significantly over time in problem-solving, depression, and destress.

^∗^Study reported significant improvement in at least one target outcome in the intervention group as compared to the control/comparison group. CESD: Center for Epidemiological Studies-Depression Scale; HADS: Hospital Anxiety and Depression Scale; CSI: Caregiver Strain Index; CQoL-I: Caregiver Quality of Life Index; CSES: Caregiver Self-Efficacy Scale; TBI: traumatic brain injury; ADHD: attention deficit hyperactivity disorder; ODD: oppositional defiant disorder; SWLS: Satisfaction with Life Scale; PSS: Perceived Stress Scale; MOS: Medical Outcomes Study; HSQ: Health Status Questionnaire; PHQ: Patient Health Questionnaire; RMBPC: Revised Memory and Behavioral Problem Checklist.

**Table 5:** Combined telephone and web interventions for caregivers of persons with chronic health conditions.

Combined telephone and web interventions (*n* = 10)					
Authors		Caregiver group	Study measures	Intervention description	Findings
Eisdorfer et al. [60]^∗^	In-person, telephone, web-based supplement (with telephone technology, local resources, online discussion groups, family conferences)	Caregivers only Family caregivers living with older adults with Alzheimer's	(i) CES-D (ii) RMBPC (iii) Researcher-Developed Likert Scaled Item on Satisfaction with Social Support	Length: 12 months, with assessments at 6, 12, & 18 months. Intervention (SET) group 1: (*n* = 75) received structural ecosystems therapy, a family intervention which identifies and targets the specific problems caregivers are experiencing, the range of usable resources available to the caregiver and their formal support systems, the range of community resources available and accessible to the family, and the capacity of the caregivers and their family to collaborate in the caregiving effort. Intervention (SET+CTIS) group 2: (*n* = 77) additionally received a computer-telephone integrated system designed to augment the therapeutic intervention by facilitating linkages of the caregivers with their family, the therapist, and supportive resources outside of the home. Attention control: (*n* = 73) participants received minimal support through nondirective, noninformative telephone calls on a similar schedule and written educational materials.	The SET alone did not have a significant effect on depression for most caregivers, except for Cuban American wives. SET+CTIS was effective in lowering caregiver depression at 6 and 18 months. The MSC showed moderate decreases for white caregiver spouses but was associated with higher depression scores for Cuban American spouses. Cuban American husbands tended to show reductions in depression for SET+CTIS but increases in depression for the other two groups.

Grover et al. [61]^∗^	Intervention group only: asynchronous (interactive web-based tailored educational modules), choice of phone or email guidance from psychologist and psychotherapist	Caregivers only Caregivers of persons with subclinical & clinical anorexia nervosa	(i) HADS (ii) Experience of Caregiving Inventory (iii) Level of Expressed Emotion Scale (iv) Accommodation and Enabling Scale for Eating Disorders (v) The Eating Disorder Symptom Impact Scale (vi) Service Utilization Questionnaire	Length: 4 months, with a 2-month follow-up postintervention. Intervention: (*n* = 34) asynchronous web-based access to a multimedia, online program over 4 months which included 8 interactive web-based tailored modules based on systemic, cognitive behavioral, and motivational interviewing frameworks. Workbooks and other materials were made available for download. Moderated message boards were also available. Weekly guidance from a therapist was available up to 20 minutes a week by phone or email. These were tailored to each caregiver's needs with support and encouragement or referrals to specific parts of the program. Usual care/waitlist: (*n* = 30) access to usual eating disorder resources (e.g., telephone hotline services [support only, no *referrals* or advice given, not necessarily the same person each time a person calls in], email support services, support groups, and moderated message board and text messaging). Provided access to the intervention after the study.	The intervention group showed more reduction in reported anxiety than the usual care group. The groups did not show significant differences in change on the other five measures.

Hicken et al. [62]^∗^	Internet or touch screen telehealth device connected through a telephone line (for subjects unfamiliar with Internet), versus telephone only	Caregivers only Family caregivers of veterans with dementia or a cognitive disorder	(i) ZBI (ii) MARWIT (sacrifice and burden, heartfelt sadness and longing, & worry and isolation subscales) (iii) PHQ (iv) 2-items (family caregiving burden causing family conflict & hardship) (v) Desire to Institutionalize Scale	Length: 4 to 6 months. Description: caregivers were stratified into 2 cohorts based upon use (*n* = 155) or nonuse (*n* = 74) of the Internet, then randomized. Intervention: (*n* = 107) electronic content (i.e., video vignettes, written information, brief assessments) on dementia progression, caregiving skills, health topics, and caregiver health were accessed 3 days per week with care manager oversight and follow-up electronically. Comparison: (*n* = 122) printed educational materials and a DVD covering content like that introduced electronically were provided, in addition to monthly telephone calls from a care manager for support.	Among home Internet users, receiving the Internet plus case management intervention predicted a decrease in reported feelings of isolation, compared to receiving the telephone only intervention. Among non-Internet users, receiving telehealth and case management predicted a decrease in reported family hardship associated with caregiving, compared to those receiving the telephone only intervention.

Mahoney et al. [63]^∗^	Telephone; a computer-mediated system using interactive voice response (IVR) with a telephone interface	Caregivers only Male and female family caregivers of older adults with Alzheimer's	(i) RMBPC (ii) CES-D (iii) SAI	Length: 12 months, with a 6-month follow-up. Intervention: (*n* = 49) received a computer-mediated system using interactive voice response (IVR) with a telephone interface. When caregivers called, the IVR queried about problem behaviors and responded with targeted information. Study personnel were alerted when a caregiver reported continuing problem behaviors and increasing stress levels during any 3-week period. The system also provided voice-mail linkage to experts in Alzheimer's and related dementias, a voice-mail telephone support group, and a distraction call for care recipients. Usual care: (*n* = 51) standard care and educational materials.	No main effects were found on any measure. In post hoc analyses, among caregivers who reported low- to mid-level mastery at baseline (*N* = 32), those in the intervention group showed a decrease in bother, depression and anxiety relative to the comparison group.

Oliver et al. [64]^∗^	Synchronous web-based videoconferencing, telephone	Caregivers only Family caregivers of adults recently enrolled in hospice with a life expectancy >2 weeks	(i) CPMQ (ii) CQoLI-R (iii) GAD-7 (iv) Pain Rating-Caregiver Report (v) Semi-Structured Interviews	Length: biweekly contacts for the duration of hospice stay (average contacts: intervention = 3.85; control = 3.98); caregiver follow-up interview 30 days after death of care recipient. Intervention: (*n* = 223) participants connected with hospice staff using telephone or online videoconferencing to discuss the care recipients' condition, identify concerns and questions, and discuss plan of care. Usual care: (*n* = 223) standard care.	The intervention group reported belief that the care recipient's pain could be managed and perceived that the care recipient's pain was better managed versus the control group. As caregiver pain management perceptions improved, caregiver anxiety significantly decreased.

Piette et al. [65]^∗^	Telephone, asynchronous web-based	Caregivers only Informal caregivers living outside the home of persons with heart failure	(i) CSI (ii) CES-D (10-item) (iii) Researcher-Developed Items regarding Time Spent Helping the Care Recipient, Involved in Transportation, and Assisting with Medications	Length: 12 months, with 6- and 12-month follow-up. Intervention: (*n* = 189) participants received weekly automated self-care support telephone calls with notifications about problems sent to clinicians, plus email summaries and suggestions for self-care assistance automatically sent to caregivers. Comparison: (*n* = 180) same treatment minus the emailed summaries and suggestions for caregivers.	Caregivers in the intervention group reported significantly less caregiving strain than those in the control at 6 and 12 months. This effect plus improvements in depressive symptoms were noted in caregivers reporting more burden at baseline. Those in the intervention group who spent the most time providing self-care support at baseline had significant decreases at both follow-ups. More caregivers increased their involvement in medical visits and medication adherence.

Powell et al. [66]^∗^	Telephone, asynchronous web-based educational modules	Caregivers only Caregivers of adults with moderate to severe TBI discharged from a level 1 trauma center	(i) BCOS (adapted for TBI (ii) BSI (iii) Life Satisfaction Scale (iv) Structured Interview (v) PART-O (vi) Modified Caregiver Appraisal Scale (caregiving mastery subscale) (vii) Caregiver Knowledge & Skill Acquisition (researcher-developed items) (viii) Brief COPE	Length: 5-months, with a 6-month follow-up. Intervention: (*n* = 77) self-guided web-based caregiving and TBI-specific online modules, plus concurrent biweekly telephone calls to discuss problems and solutions. Modules were not presented in a specific order, rather caregivers were directed to relevant modules during telephone calls as indicated by their answers to a brief interview at the onset of the call. Usual care: (*n* = 76) standard care.	Caregivers in the intervention group showed significantly higher quality of life and emotional well-being. They also showed significantly more active coping strategies and less emotional venting coping styles at follow-up. Caregivers also tended to report more support from others, feeling more competent at obtaining information from medical professionals, and taking care of themselves than usual care.

Steel et al. [67]^∗^	Telephone, asynchronous website, in-person (intervention only)	Caregiver-care recipient dyads Family caregivers of persons with advanced cancer	(i) CES-D (ii) Caregiver Quality of Life Index-Cancer Scale	Length: 6 months. Intervention: (*n* = 124 dyads) access to a web site with written and audiovisual self-management strategies, a bulletin board, and other resources; visits with a care coordinator every 2 months during a physician visit; and telephone follow-up every 2 weeks. Participants also received visits from a care coordinator. Usual care: (*n* = 100 dyads) standard care.	Caregiver stress and depression decreased significantly at 6 months for those in the intervention group.

Steffen and Gant [68]^∗^	Telephone, videos (intervention), mailed information booklets (comparison)	Caregivers only Women caring for older adults with neurocognitive disorders	(i) RMBC (ii) BDI II (iii) Negative Affect Scale (iv) MAACL-R (anxiety & hostility subscales) (v) Caregiving Self-Efficacy-Revised (obtaining respite & responding to disruptive behaviors subscales)	Length: 14 weeks; follow-up assessments conducted by interviewers at preintervention, postintervention, and 6 months. Intervention: (*n* = 33) behavioral coaching using online video instructional materials, workbook, and 12 telephone coaching sessions (10 weekly, last 2 biweekly) in behavioral management, pleasant events scheduling, and relaxation. Attention control: (*n* = 41) basic education guide and telephone support in 7 bimonthly calls.	Depressive symptoms, upset following disruptive behaviors, and negative mood states were statistically lower in the intervention group than in the comparison group posttreatment. Reliable change index analyses for BDI II scores favored the intervention. Caregiving self-efficacy scores for obtaining respite and for managing patient behavioral disturbances were significantly higher.

Williams et al. [69]^∗^	Stand-alone video series, telephone	Caregivers only Family caregivers of older adults with Alzheimer's disease or related dementia	(i) Perceived Stress Scale (ii) State Trait Anxiety Inventory (iii) State-Trait Anger Inventory (iv) CES-D (v) Cook-Medley Hostility Scale (vi) Caregiving Self-Efficacy-Revised (vii) Pittsburgh Sleep Quality Index (viii) Blood Pressure (ix) Heart Rate (x) Salivary Cortisol Levels	Length: 5 weeks, with follow-up 4 1/2 months postintervention. Intervention: (*n* = 59) online video-based modules with coping skills training related to dementia caregiving, with an accompanying workbook, and 5 weekly telephone coaching sessions. Wait list control: (*n* = 57) received the intervention following the study.	Outcomes for the intervention and wait list control groups significantly differed on depression, perceived stress, and blood pressure. Differences in the predicted direction were sustained through follow-up assessments at 5 months postintervention, but interpretability of these findings is limited by baseline differences in some variables.

Study reported significant improvement in at least one target outcome in the intervention group as compared to the control/comparison group. MARWIT: Marwit-Meuser Caregiver Grief Inventory-Short Form; CSI: Caregiver Strain Index; CQoLI-R: Caregiver Quality of Life Index-Revised; GAD-7: Generalized Anxiety Dixorder-7; TBI: traumatic brain injury; BSI: Brief Symptom Inventory; PART-O: Participation Assessment with Recombined Tools-Objective; RMBC: Revised Memory & Behavior Problems Checklist; BDI II: Beck Depression Inventory II; MAACL-R: Multiple Affect Adjective Check List-Revised; ZBI: Zarit Burden Interview; SAI: State Anxiety Inventory; PHQ: Patient Health Questionnaire; BCOS: Bakas Caregiving Outcomes Scale; HADS: Hospital Anxiety and Depression Scale; RMBPC: Revised Memory and Behavioral Problem Checklist.

**(a):** 

Telephone interventions (*n* = 33)													
Authors	Caregiver intervention components					Caregiver applications						User control	
	Skills training group (G) or individual (I)	Psychoeducation & resource materials	Self-monitoring/tracking	Reminders	Counseling group (G) or individual (I)	Email	Discussion forum/online chat	Online journal	Text message	Telephone calls^∗∗^	Useful links & resources	Facilitator-guided	Self-guided
Au et al. [13]	X (I)	X			X (I)							X	
Bakas et al. [14]	X (I)	X	X									X	
Bakas et al. [15]	X (I)	X										X	
Campbell et al. [16]	X (G)		X									X	
Chodosh et al. [17]	X (I)	X			X(I)							X	
Davis et al. [18]	X (I)	X	X									X	
Demiris et al. [19]	X (I)											X	
Dionne-Odom et al. [20]	X (I)											X	
Dollinger and Chwalisz [21]	X (I)									X		X	
Elliott et al. [22]	X (I)	X										X	
Elliott and Berry [23]	X (I)											X	
Farran et al. [24]	X (G) & (I)											X	
Farran et al. [25]	X (I)		X									X	
Finkel et al. [26]	X (I)				X (G)							X	
Gant et al. [27]	X (I)	X	X									X	
Grant et al. [28]	X (I)		X									X	
Hartke and King [29]		X			X (G)							X	
Hu et al. [30]	X (G) & (I)									X		X	
Keeping-Burke et al. [31]		X			X (I)							X	
King et al. [32]	X (I)		X									X	
Kwok et al. [33]	X (I)	X										X	
Lingler et al. [34]	X (I)	X										X	
McCann et al. [35]		X								X			X
Porter et al. [36]	X (G)	X										X	
Rivera et al. [37]	X (I)	X										X	
Shaw et al. [38]					X (I)							X	
Smith and Toseland [39]	X (G)	X			X (G)					X		X	
Tanner et al. [40]	X (I)	X								X		X	
Tremont et al. [41]	X (I)	X		X								X	
Tremont et al. [42]		X			X (I)							X	
Wilz et al. [43]	X (I)											X	
Wilz and Soellner et al. [44]	X (I)	X			X (I)							X	
Winter and Gitlin [45]		X			X (G)								X

**(b):** 

Web interventions (*n* = 14)													
Authors	Caregiver intervention components					Caregiver applications						User control	
	Skills training group (G) or individual (I)	Psychoeducation & resource materials	Self-monitoring/tracking	Reminders	Counseling group (G) or individual (I)	Email	Discussion forum/online chat	Online journal	Text message	Telephone calls^∗∗^	Useful links & resources	Facilitator-guided	Self-guided
Beauchamp et al. [46]	X (I)	X											X
Blom et al. [47]	X (I)	X		X		X							X
Chih et al. [48]	X (I)	X	X	X		X	X				X		X
Hattink et al. [49]	X (I)	X					X				X		X
Kajiyama et al. [50]	X (I)	X											X
Klemm et al. [51]					X (G)		X					X	
Marziali and Donahue [52]		X			X (G)	X	X				X	X	
Petranovich et al. [53]	X (G)												X
Pierce et al. [54]						X				X	X		X
Raj et al. [55]	X (G)	X									X	X	
Smith et al. [56]	X (G)	X	X			X	X				X	X	
Vander Stoep et al. [57]	X (G)	X										X	
Wade et al. [58]	X (G)	X									X		X
Wade et al. [59]	X (G)	X									X	X	

**(c):** 

Combined telephone and web interventions (*n* = 10)													
Authors	Caregiver intervention components					Caregiver applications						User control	
	Skills training group (G) or individual (I)	Psychoeducation & resource materials	Self-monitoring/tracking	Reminders	Counseling group (G) or individual (I)	Email	Discussion forum/online chat	Online journal	Text message	Telephone calls^∗∗^	Useful links & resources	Facilitator-guided	Self-guided
Eisdorfer et al. [60]		X			X (G)		X				X	X	
Grover et al. [61]		X			X (I)	X	X						X
Hicken et al. [62]	X (I)	X	X							X	X		X
Mahoney et al. [63]	X (I)	X	X				X						X
Oliver et al. [64]			X									X	
Piette et al. [65]		X	X	X		X				X	X		X
Powell et al. [66]	X (I)	X										X	
Steel et al. [67]		X	X		X (I)		X	X		X	X	X	
Steffen and Grant [68]	X (I)	X										X	
Williams et al. [69]	X (I)	X										X	

## Data Availability

For information regarding the data supporting the results of this study, please email lgraven@fsu.edu.

## References

[1] National Alliance for Caregiving (NAC) Caregiving in the U.S.. https://www.aarp.org/content/dam/aarp/ppi/2015/caregiving-in-the-united-states-2015-report-revised.pdf/.

[2] Glueckauf R. L., Noël L. T., Toseland R. W., Haigler D. H., Monahan D. J. (2011). Telehealth and family caregiving: developments in research, education, policy, and practice. *Education and Support Programs for Caregivers*.

[3] Alzheimer’s Association (2018). Alzheimer’s disease facts and figures. *Alzheimer’s & Dementia*.

[4] Wells B. A., Glueckauf R. L., Bernabe D. (2017). African American dementia caregiver problem inventory: descriptive analysis and initial psychometric evaluation. *Rehabilitation Psychology*.

[5] Joling K. J., van Marwijk H. W., Veldhuijzen A. E. (2015). The two-year incidence of depression and anxiety disorders in spousal caregivers of persons with dementia: who is at the greatest risk?. *The American Journal of Geriatric Psychiatry*.

[6] House of Representatives (111th Congress) (2010). National Alzheimer’s Project Act of 2010. S. 3036. https://www.govtrack.us/congress/bills/111/hr4689/text/.

[7] United States Department of Veteran Affairs (2019). REACH VA Program. https://www.caregiver.va.gov/REACH_VA_Program.asp/.

[8] Rosalyn Carter Institute for Caregiving (2019). Operation family caregiver. http://www.operationfamilycaregiver.org/.

[9] Ehde D. M., Dillworth T. M., Turner J. A. (2014). Cognitive-behavioral therapy for individuals with chronic pain: efficacy, innovations, and directions for research. *The American Psychologist*.

[10] Glueckauf R. L., Davis W. S., Willis F. (2012). Telephone-based, cognitive-behavioral therapy for African American dementia caregivers with depression: initial findings. *Rehabilitation Psychology*.

[11] Chi N., Demiris G. (2015). A systematic review of telehealth tools and interventions to support family caregivers. *Journal of Telemedicine and Telecare*.

[12] AHRQ (2019). ePSS. https://epss.ahrq.gov/PDA/index.jsp/.

[13] Au A., Gallagher-Thompson D., Wong M. (2015). Behavioral activation for dementia caregivers: scheduling pleasant events and enhancing communications. *Clinical Interventions in Aging*.

[14] Bakas T., Farran C. J., Austin J. K., Given B. A., Johnson E. A., Williams L. S. (2009). Stroke caregiver outcomes from the telephone assessment and skill-building kit (TASK). *Topics in Stroke Rehabilitation*.

[15] Bakas T., Austin J. K., Habermann B. (2015). Telephone assessment and skill-building kit for stroke caregivers: a randomized controlled clinical trial. *Stroke*.

[16] Campbell L. C., Keefe F. J., Scipio C. (2007). Facilitating research participation and improving quality of life for African American prostate cancer survivors and their intimate partners. *Cancer*.

[17] Chodosh J., Colaiaco B. A., Connor K. I. (2015). Dementia care management in an underserved community: the comparative effectiveness of two different approaches. *Journal of Aging and Health*.

[18] Davis L. L., Burgio L. D., Buckwalter K. C., Weaver M. (2004). A comparison of in-home and telephone-based skill training interventions with caregivers of persons with dementia. *Journal of Mental Health and Aging*.

[19] Demiris G., Parker Oliver D., Wittenberg-Lyles E. (2012). A noninferiority trial of a problem-solving intervention for hospice caregivers: in person versus videophone. *Journal of Palliative Medicine*.

[20] Dionne-Odom J. N., Azuero A., Lyons K. D. (2016). Family caregiver depressive symptom and grief outcomes from the ENABLE III randomized controlled trial. *Journal of Pain and Symptom Management*.

[21] Clancy Dollinger S., Chwalisz K. (2011). Reaching rural caregivers with a multicomponent telehealth intervention: the telehelp line for caregivers. *Professional Psychology: Research and Practice*.

[22] Elliott T. R., Brossart D., Berry J. W., Fine P. R. (2008). Problem-solving training via videoconferencing for family caregivers of persons with spinal cord injuries: a randomized controlled trial. *Behaviour Research and Therapy*.

[23] Elliott T. R., Berry J. W. (2009). Brief problem-solving training for family caregivers of persons with recent-onset spinal cord injuries: a randomized controlled trial. *Journal of Clinical Psychology*.

[24] Farran C. J., Gilley D. W., McCann J. J., Bienias J. L., Lindeman D. A., Evans D. A. (2004). Psychosocial interventions to reduce depressive symptoms of dementia caregivers: a randomized clinical trial comparing two approaches. *Journal of Mental Health and Aging*.

[25] Farran C., Paun O., Cothran F. (2015). Impact of an individualized physical activity intervention on improving mental health outcomes in family caregivers of persons with dementia: a randomized controlled trial. *AIMS Medical Science*.

[26] Finkel S., Czaja S. J., Martinovich Z., Harris C., Pezzuto D., Schulz R. (2007). E-Care: a telecommunications technology intervention for family caregivers of dementia patients. *The American Journal of Geriatric Psychiatry*.

[27] Gant J. R., Steffen A. M., Lauderdale S. A. (2007). Comparative outcomes of two distance-based interventions for male caregivers of family members with dementia. *American Journal of Alzheimer’s Disease and Other Dementias*.

[28] Grant J. S., Elliott T. R., Weaver M., Bartolucci A. A., Giger J. N. (2002). Telephone intervention with family caregivers of stroke survivors after rehabilitation. *Stroke*.

[29] Hartke R. J., King R. B. (2003). Telephone group intervention for older stroke caregivers. *Topics in Stroke Rehabilitation*.

[30] Hu X., Dolansky M. A., Su Y., Hu X., Qu M., Zhou L. (2016). Effect of a multidisciplinary supportive program for family caregivers of patients with heart failure on caregiver burden, quality of life, and depression: a randomized controlled study. *International Journal of Nursing Studies*.

[31] Keeping-Burke L., Purden M., Frasure-Smith N., Cossette S., McCarthy F., Amsel R. (2013). Bridging the transition from hospital to home: effects of the VITAL telehealth program on recovery for CABG surgery patients and their caregivers. *Research in Nursing & Health*.

[32] King A. C., Baumann K., O'Sullivan P., Wilcox S., Castro C. (2002). Effects of moderate-intensity exercise on physiological, behavioral, and emotional responses to family caregiving: a randomized controlled trial. *The Journals of Gerontology. Series A, Biological Sciences and Medical Sciences*.

[33] Kwok T., Wong B., Ip I., Chui K., Young D., Ho F. (2013). Telephone-delivered psychoeducational intervention for Hong Kong Chinese dementia caregivers: a single-blinded randomized controlled trial. *Clinical Interventions in Aging*.

[34] Lingler J. H., Sereika S. M., Amspaugh C. M. (2016). An intervention to maximize medication management by caregivers of persons with memory loss: intervention overview and two-month outcomes. *Geriatric Nursing*.

[35] McCann T. V., Songprakun W., Stephenson J. (2015). A randomized controlled trial of guided self-help for improving the experience of caring for carers of clients with depression. *Journal of Advanced Nursing*.

[36] Porter L. S., Keefe F. J., Garst J. (2011). Caregiver-assisted coping skills training for lung cancer: results of a randomized clinical trial. *Journal of Pain and Symptom Management*.

[37] Rivera P. A., Elliott T. R., Berry J. W., Grant J. S. (2008). Problem-solving training for family caregivers of persons with traumatic brain injuries: a randomized controlled trial. *Archives of Physical Medicine and Rehabilitation*.

[38] Shaw J. M., Young J. M., Butow P. N. (2016). Improving psychosocial outcomes for caregivers of people with poor prognosis gastrointestinal cancers: a randomized controlled trial (Family Connect). *Supportive Care in Cancer*.

[39] Smith T. L., Toseland R. W. (2006). The effectiveness of a telephone support program for caregivers of frail older adults. *The Gerontologist*.

[40] Tanner J. A., Black B. S., Johnston D. (2015). A randomized controlled trial of a community-based dementia care coordination intervention: effects of MIND at Home on caregiver outcomes. *The American Journal of Geriatric Psychiatry*.

[41] Tremont G., Duncan Davis J., Bishop D. S., Fortinsky R. H. (2008). Telephone-delivered psychosocial intervention reduces burden in dementia caregivers. *Dementia*.

[42] Tremont G., Davis J. D., Papandonatos G. D. (2015). Psychosocial telephone intervention for dementia caregivers: a randomized controlled trial. *Alzheimer’s & Dementia*.

[43] Wilz G., Schinkӧthe D., Soellner R. (2011). Goal attainment and treatment compliance in a cognitive-behavioral telephone intervention for family caregivers of persons with dementia. *GeroPsych*.

[44] Wilz G., Soellner R. (2016). Evaluation of a short-term telephone-based cognitive behavioral intervention for dementia family caregivers. *Clinical Gerontologist*.

[45] Winter L., Gitlin L. N. (2007). Evaluation of a telephone-based support group intervention for female caregivers of community-dwelling individuals with dementia. *American Journal of Alzheimer’s Disease and Other Dementias*.

[46] Beauchamp N., Irvine A. B., Seeley J., Johnson B. (2005). Worksite-based Internet multimedia program for family caregivers of persons with dementia. *The Gerontologist*.

[47] Blom M. M., Zarit S. H., Groot Zwaaftink R. B. M., Cuijpers P., Pot A. M. (2015). Effectiveness of an Internet intervention for family caregivers of people with dementia: results of a randomized controlled trial. *PLoS One*.

[48] Chih M., DuBenske L. L., Hawkins R. P. (2013). Communicating advanced cancer patients’ symptoms via the Internet: a pooled analysis of two randomized trials examining caregiver preparedness, physical burden, and negative mood. *Palliative Medicine*.

[49] Hattink B., Meiland F., van der Roest H. (2015). Web-based STAR E-Learning course increases empathy and understanding in dementia caregivers: results from a randomized controlled trial in the Netherlands and the United Kingdom. *Journal of Medical Internet Research*.

[50] Kajiyama B., Thompson L. W., Eto-Iwase T. (2013). Exploring the effectiveness of an Internet-based program for reducing caregiver distress using the iCare Stress Management e-Training Program. *Aging & Mental Health*.

[51] Klemm P., Hayes E., Diefenbeck C., Milcarek B. (2014). Online support for employed informal caregivers. *Computers, Informatics, Nursing*.

[52] Marziali E., Donahue P. (2006). Caring for others: Internet video-conferencing group intervention for family caregivers of older adults with neurodegenerative disease. *The Gerontologist*.

[53] Petranovich C. L., Wade S. L., Taylor H. G. (2015). Long-term caregiver mental health outcomes following a predominately online intervention for adolescents with complicated mild to severe traumatic brain injury. *Journal of Pediatric Psychology*.

[54] Pierce L. L., Steiner V. L., Khuder S. A., Govoni A. L., Horn L. J. (2009). The effect of a web-based stroke intervention on carers’ well-being and survivors’ use of healthcare services. *Disability and Rehabilitation*.

[55] Raj S. P., Antonini T. N., Oberjohn K. S., Cassedy A., Makoroff K. L., Wade S. L. (2015). Web-based parenting skills program for pediatric traumatic brain injury reduces psychological distress among lower-income parents. *The Journal of Head Trauma Rehabilitation*.

[56] Smith G. C., Egbert N., Dellman-Jenkins M., Nanna K., Palmieri P. A. (2012). Reducing depression in stroke survivors and their informal caregivers: a randomized clinical trial of a web-based intervention. *Rehabilitation Psychology*.

[57] Vander Stoep A., McCarty C. A., Zhou C., Rockhill C. M., Schoenfelder E. N., Myers K. (2017). The children’s attention-deficit hyperactivity disorder telemental health treatment study: caregiver outcomes. *Journal of Abnormal Child Psychology*.

[58] Wade S. L., Carey J., Wolfe C. R. (2006). An online family intervention to reduce parental distress following pediatric brain injury. *Journal of Consulting and Clinical Psychology*.

[59] Wade S. L., Walz N. C., Carey J. (2012). A randomized trial of teen online problem solving: efficacy in improving caregiver outcomes after brain injury. *Health Psychology*.

[60] Eisdorfer C., Czaja S. J., Loewenstein D. A. (2003). The effect of a family therapy and technology-based intervention on caregiver depression. *The Gerontologist*.

[61] Grover M., Naumann U., Mohammad-Dar L. (2011). A randomized controlled trial of an Internet-based cognitive-behavioural skills package for carers of people with anorexia nervosa. *Psychological Medicine*.

[62] Hicken B. L., Daniel C., Luptak M., Grant M., Kilian S., Rupper R. W. (2017). Supporting caregivers of rural veterans electronically (SCORE). *The Journal of Rural Health*.

[63] Mahoney D. F., Tarlow B. J., Jones R. N. (2003). Effects of an automated telephone support system on caregiver burden and anxiety: findings from the REACH for TLC intervention study. *The Gerontologist*.

[64] Parker Oliver D., Demiris G., Washington K., Kruse R. L., Petroski G. (2017). Hospice family caregiver involvement in care plan meetings: a mixed-methods randomized controlled trial. *The American Journal of Hospice & Palliative Care*.

[65] Piette J. D., Striplin D., Marinec N., Chen J., Aikens J. E. (2015). A randomized trial of mobile health support for heart failure patients and their informal caregivers. *Medical Care*.

[66] Powell J. M., Fraser R., Brockway J. A., Temkin N., Bell K. R. (2016). A telehealth approach to caregiver self-management following traumatic brain injury. *The Journal of Head Trauma Rehabilitation*.

[67] Steel J. L., Geller D. A., Kim K. H. (2016). Web-based collaborative care intervention to manage cancer-related symptoms in the palliative care setting. *Cancer*.

[68] Steffen A. M., Gant J. R. (2016). A telehealth behavioral coaching intervention for neurocognitive disorder family carers. *International Journal of Geriatric Psychiatry*.

[69] Williams V. P., Bishop-Fitzpatrick L., Lane J. D. (2010). Video-based coping skills to reduce health risk and improve psychological and physical well-being in Alzheimer’s disease family caregivers. *Psychosomatic Medicine*.

[70] Glueckauf R. L., Loomis J. S. (2003). Alzheimer’s caregiver support online: lessons learned, initial findings and future directions. *NeuroRehabilitation*.

